# Two‐Dimensional Polycyclodextrins for Strong Multivalent Host‐Guest Interactions at Biointerfaces

**DOI:** 10.1002/smll.202412282

**Published:** 2025-04-27

**Authors:** Zahra Goudarzi, Zahra Mohammadi, Reza Maleki, Siamak Beyranvand, Chuanxiong Nie, Mohammad Fardin Gholami, Özge Akkaya, Mahdieh Kalantari, Mohammad Nemati, Fatemeh Yousufvand, Fatemeh Shahverdi, Marzieh Rashidipour, Zainab Ahmadian, Ievgen Donskyi, Philip Nickl, Marek Brzeziński, Kai Ludwig, Jürgen P. Rabe, Raul Arenal, Cheng Chong, Angelo H. ALL, Mohsen Adeli

**Affiliations:** ^1^ Department of Chemistry Lorestan University Khorramabad 6815144316 Iran; ^2^ Department of Chemical Technologies Iranian Research Organization for Science and Technology (IROST) Tehran 33535111 Iran; ^3^ Institut für Chemie und Biochemie Freie Universität Berlin Takustr. 3 14195 Berlin Germany; ^4^ Department of Physics & IRIS Adlershof Humboldt‐Universität zu Berlin Newtonstrasse 15 12489 Berlin Germany; ^5^ Environmental Health Research Center Lorestan University of Medical Sciences Khorramabad 6816889468 Iran; ^6^ Department of Pharmaceutics School of Pharmacy Lorestan University of Medical Sciences Khorramabad 6815144311 Iran; ^7^ BAM – Federal Institute for Material Science and Testing, Division of Surface Analysis and Interfacial Chemistry Unter den Eichen 44–46 12205 Berlin Germany; ^8^ Centre of Molecular and Macromolecular Studies Polish Academy of Sciences Sienkiewicza 112 Łódź 90‐363 Poland; ^9^ Forschungszentrum für Elektronenmikroskopie and Core Facility BioSupraMol Institut für Chemie und Biochemie Freie Universität Berlin Fabeckstr. 36a 14195 Berlin Germany; ^10^ Instituto de Nanociencia y Materiales de Aragon (INMA) CSIC‐Universidad de Zaragoza Zaragoza 50009 Spain; ^11^ Laboratorio de Microscopias Avanzadas (LMA) Universidad de Zaragoza Zaragoza 50018 Spain; ^12^ Fundacion ARAID Zaragoza 50018 Spain; ^13^ College of Polymer Science and Engineering State Key Laboratory of Polymer Materials Engineering Sichuan University Chengdu 610065 China; ^14^ Department of Chemistry Hong Kong Baptist University Hong Kong SAR 999077 China

**Keywords:** 2D polymers, atherosclerosis, multivalent host‐guest, polycyclodextrins, virus inhibition

## Abstract

While 2D polymers with aromatic backbones have been increasingly receiving interest from various scientific disciplines, their nonaromatic counterparts are less investigated. In this work, 2D poly(*β*‐cyclodextrin)s (2D‐CDs) with few hundred nanometers to millimeters lateral sizes and 0.7 nm thickness are synthesized using graphene and boron nitride as colloidal templates and used for multivalent host‐guest interactions with biological systems. Deposition of cyclodextrins on graphene and boron nitride templates followed by lateral crosslinking and template detachment resulted in 2D‐CDs with different physicochemical properties. The size of the 2D‐CDs is dominated by noncovalent interactions between cyclodextrins and templates. While an interaction energy of −224.3 kJ mol^−1^ at the interface between graphene and cyclodextrin led to few hundred nanometer 2D‐CDs, boron nitride with weaker interactions (−179.4 kJ mol^−1^) resulted in polymers with millimeters lateral sizes. The secondary hydroxyl groups of 2D‐CDs are changed to sodium sulfate, and 2D polymers with the ability of simultaneous host‐guest and electrostatic interactions with biosystems including vessel plaques and herpes simplex virus (HSV) are obtained. The sulfated 2D‐CDs (2D‐CDSs) show a high ability for virus binding (IC50 = 6 µg mL^−1^). Owing to their carbohydrate backbone, 2D‐CDs are novel heparin mimetics that can be formulated for efficient inhibition of viral infections.

## Graphical abstract

Two‐dimensional poly(β‐cyclodextrin)s (2D‐CDs) are synthesized using graphene and boron nitride templates to create nanosheets capable of multivalent host‐guest interactions at biointerfaces. Template‐driven assembly controls sheet size, while sulfation enhances electrostatic interactions at biointerfaces. The resulting 2D‐CDs bind biological targets with high affinity, acting as carbohydrate‐based, biomimicry platforms. These nonaromatic 2D polymers showcase the power of multivalency for selective, strong binding in biomedical applications.

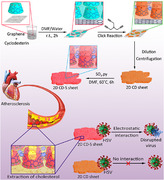

## 1 Introduction

2D polymers have emerged as a new class of macromolecules with unique optoelectronic, physicochemical, mechanical, and biological properties.^[^ ^1^, ^2^, ^3^, ^4^, ^5^, ^6^, ^7^, ^8^, ^9^, ^10^ ^]^ They are excellent candidates for biomedical applications, for example, tissue engineering, cancer therapy and pathogen interactions, because of high aspect ratio and defined structures that give rise to the high loading capacity and specific interactions at biointerfaces.^[^ ^11^, ^12^, ^13^, ^14^, ^15^ ^]^

Confining monomers in a sheet‐like structure and performing polymerizations in 2D, however, is energetically unfavorable and causes many challenges in the construction of 2D polymers.^[^ ^1^, ^16^, ^17^, ^18^, ^19^, ^20^, ^21^ ^]^ A common procedure to constrain monomers into a sheet‐like structure during or prior to polymerization is to use driving forces at an interface.^[^ ^2^, ^19^, ^22^, ^23^, ^24^, ^25^ ^]^ Depending on their structures and functional groups, monomers can create supramolecular mono‐ or multilayers at different interfaces, which can be polymerized into a 2D polymer using a suitable initiator.^[^ ^21^, ^26^, ^27^, ^28^, ^29^, ^30^ ^]^ Driving polymerizations in 2D using a platform at solid/liquid and solid/air interfaces is called template‐assisted strategy.^[^ ^31^, ^32^, ^33^ ^]^ Colloidal templates dispersible in aqueous and organic solvents have recently been used to confine monomers in a sheet‐like supramolecular structure and synthesize 2D polymers.^[^ ^34^ ^]^ This method takes advantages of driving forces at both solid and solution states. Since the interactions between templates and monomers are dominated by the solid/liquid microenvironment and the entire system, including templates, monomers and reagents are dispersed in a solvent as a reaction environment,^[^ ^35^ ^]^ such a system enables us to control 2D polymerization by manipulation of the thermodynamic and kinetic parameters of both reaction microenvironment and environment. The colloidal templates provide new opportunities for the development of a variety of 2D polymers, which are hardly achievable by other methods. Another challenge with the current 2D polymerizations is that they are limited to aromatic monomers.^[^ ^36^, ^37^ ^]^ To open up new avenues for the preparation of biocompatible 2D polymers, for example polysaccharides, for future biomedical applications, new strategies for the polymerization of non‐aromatic monomers are required.^[^ ^12^, ^38^ ^]^ Recently, we reported a new functionalization method for graphene sheets that allows the preparation of colloidal templates with defined functionality at ambient conditions.^[^ ^39^, ^40^, ^41^, ^42^, ^43^ ^]^ We used this colloidal templates for the production of 2D polyglycerols with a lateral size of a few hundred nanometers.^[^ ^34^ ^]^ It is well‐documented that graphene and boron nitride templates, dispersed in aqueous solutions, are able to load small molecules by π‐π stacking and hydrophobic or van der Waals interactions. These interactions can be used for loading and lateral crosslinking of carbohydrate monomers which are not supported with a π system.^[^ ^41^, ^44^, ^45^, ^46^ ^]^

In this work, 2D poly(*β*‐cyclodextrin)s (2D‐CDs) with a lateral size ranging from hundreds of nanometers to millimeters were synthesized using colloidal graphene and boron nitride templates. While strong interactions between cyclodextrin monomers and a graphene template led to 2D‐CDs with a lateral size of a few hundred nanometers, weak interactions between boron nitride and cyclodextrin resulted in their counterparts in the range of several micrometers to millimeters. Moreover, the hydroxyl groups of the wider rim of 2D‐CDs were changed to sodium sulfate to mimic the extracellular matrix for virus blocking and inactivation. The sulfated 2D‐CDs (2D‐CDSs) were able to interact strongly with human herpes simplex virus (HSV) and effectively protect cells from infection. Moreover, 2D‐CDSs were able to extract cholesterol from vascular plaques by multivalent host‐guest interactions opening up new avenues for future biomedical applications.

## 2 Results and Discussion

The template‐assisted strategy is a well‐studied and efficient way for the construction of 2D polymers. Noncovalent interactions between monomers and templates are effective driving forces to confine monomers at this interface, rendering polymerization of multifunctional monomers in sheet‐like structures.

In this work, thermally reduced graphene oxide (rGO) and boron nitride (BN) were used as templates to drive polymerization of functional cyclodextrins in 2D. Templates with a lateral size of several micrometers (Figure S2, Supporting Information) were supposed to load cyclodextrins in aqueous solutions and organize them into layered structures.

**Figure 1:**
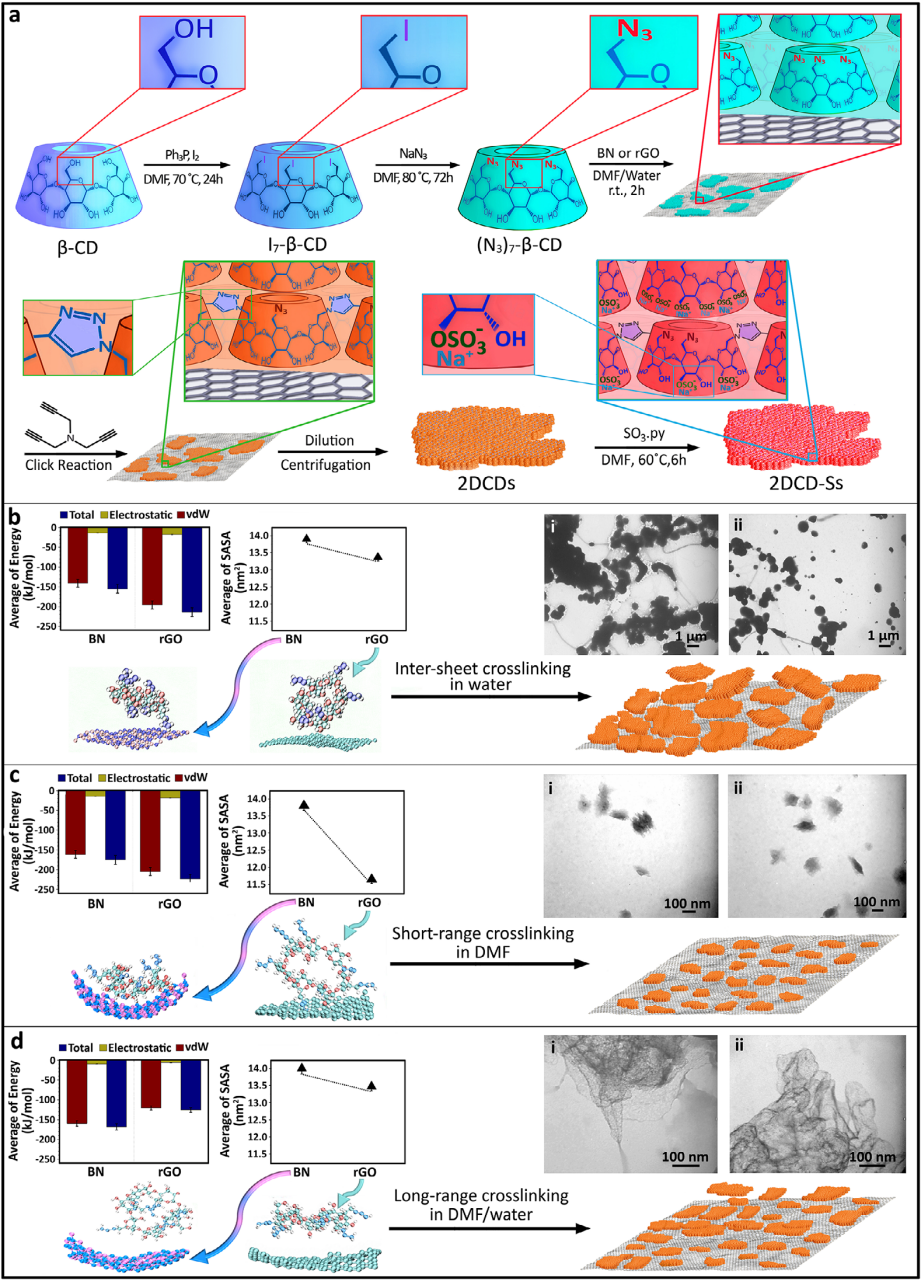
a) Schematic representation of the synthesis of 2D poly(*β*‐cyclodextrin)s and sulfation of their secondary hydroxyl functional groups to boost their multivalent host‐guest interactions with biosystems by electrostatic forces. Primary functional groups of *β*‐cyclodextrin were changed to azide and heptakis‐(6‐azido‐6‐deoxy)‐*β*‐cyclodextrin as a multifunctional monomer was prepared for the lateral crosslinking on templates. b) total energy interactions between rGO and BN templates and monomers in water were −224.3 and −179.4 kJ mol^−1^, respectively. While such interactions were in a suitable range, low dispersibility of templates in water caused inter‐sheet crosslinking and resulted in irregular 3D objects. Transmission electron microscopy (TEM) images of the product of reactions on rGO and BN templates (i and ii respectively) indicated 3D structures. c) Simulation studies showed −210.9 and −157.6 kJ mol^−1^ total energy interactions between rGO and BN templates and monomers in DMF, respectively. Interactions between templates and monomers were too strong and inhibited long‐range lateral growing of 2D poly(*β*‐cyclodextrin)s. TEM images of the product of reaction on rGO and BN templates (i and ii respectively) showed small sheets with a lateral size of ≈100 nm. d) DMF/water mixture was used as an optimum system for the polymerization of heptakis‐(6‐azido‐6‐deoxy)‐*β*‐cyclodextrin on templates. In DMF/water mixture dispersibility of templates and mobility of monomers were balanced for an efficient lateral crosslinking of monomers. TEM images of the product of reaction on rGO and BN templates (i and ii respectively) demonstrated layered structures with a lateral size of several micrometers.

The primary hydroxyl functional groups of *β*‐cyclodextrin were changed to azide and subjected to a lateral crosslinking via copper‐catalyzed click reaction using tripropargyl amine as a crosslinker (**Figure** **1**a).

In order to explore the ability of the graphene template to load and confine cyclodextrin monomers in supramolecular monolayers, the noncovalent interactions between freshly cleaved highly oriented pyrolytic graphite (HOPG) and heptakis‐(6‐azido‐6‐deoxy)‐*β*‐cyclodextrin (*β*‐CD‐N_3_) were investigated in dimethylformamide (DMF) solvent.

Figure S3 (Supporting Information) demonstrates the typical Scanning Force Microscopy (SFM)‐Quantitative Imaging Mode height overview of a freshly cleaved HOPG surface at ambient conditions before and after addition of DMF solution containing *β*‐CD‐N_3_. Thin layers of *β*‐CD‐N_3_ were observed over the HOPG liquid/solid interface, which were not observed in a blank solution of DMF. The height of the layers formed on the flat terraces of HOPG was 0.79 nm, which is consistent with the theoretical height of *β*‐CD (0.78 nm)^[^ ^47^ ^]^ and expected for *β*‐CD‐N_3_, considering azide functional groups.

The process of assembly of *β*‐CD‐N_3_ on HOPG was dynamic and resulted in cyclodextrin islands in 5–20 min. Moreover, the rate of growth of the layers on HOPG was inversely affected by the scanning speed. Regardless of differences between the chemical structure of HOPG and rGO, in terms of defects and crystalline domains, the ability of HOPG to create monolayers of *β*‐CD‐N_3_ indicated suitability of graphene as a template for confining cyclodextrin monomers in sheet‐like structures in DMF. Noncovalent interactions between *β*‐CD‐N_3_ and templates including rGO and BN played a dual role in 2D polymerization of this monomer. *β*‐CD‐N_3_ supported dispersion of templates in solvents and templates confined the loaded monomers in 2D simultaneously.

In addition to intrinsic advantages including low toxicity and environmentally friendly features, purification and separation of poly(*β*‐cyclodextrin) from templates in water was a straightforward process. Owing to such significant aspects, water was selected as the solvent of polymerization of *β*‐CD‐N_3_ on the surface of templates. Simulation studies indicated −224.3 and −179.4 kJ mol^−1^ total energy interactions between rGO and BN templates and monomers in water, respectively (Figure 1b). Van der Waals interactions were the main forces, and hydrophobic interactions didn't play a significant role at this interface. Template‐assisted polymerization of monomers in water, however, resulted in irregular materials (Figure 1b). Low dispersity and accumulation of templates in water caused an inter‐sheet crosslinking and production of irregular 3D objects (Figure 1b).

**Figure 2:**
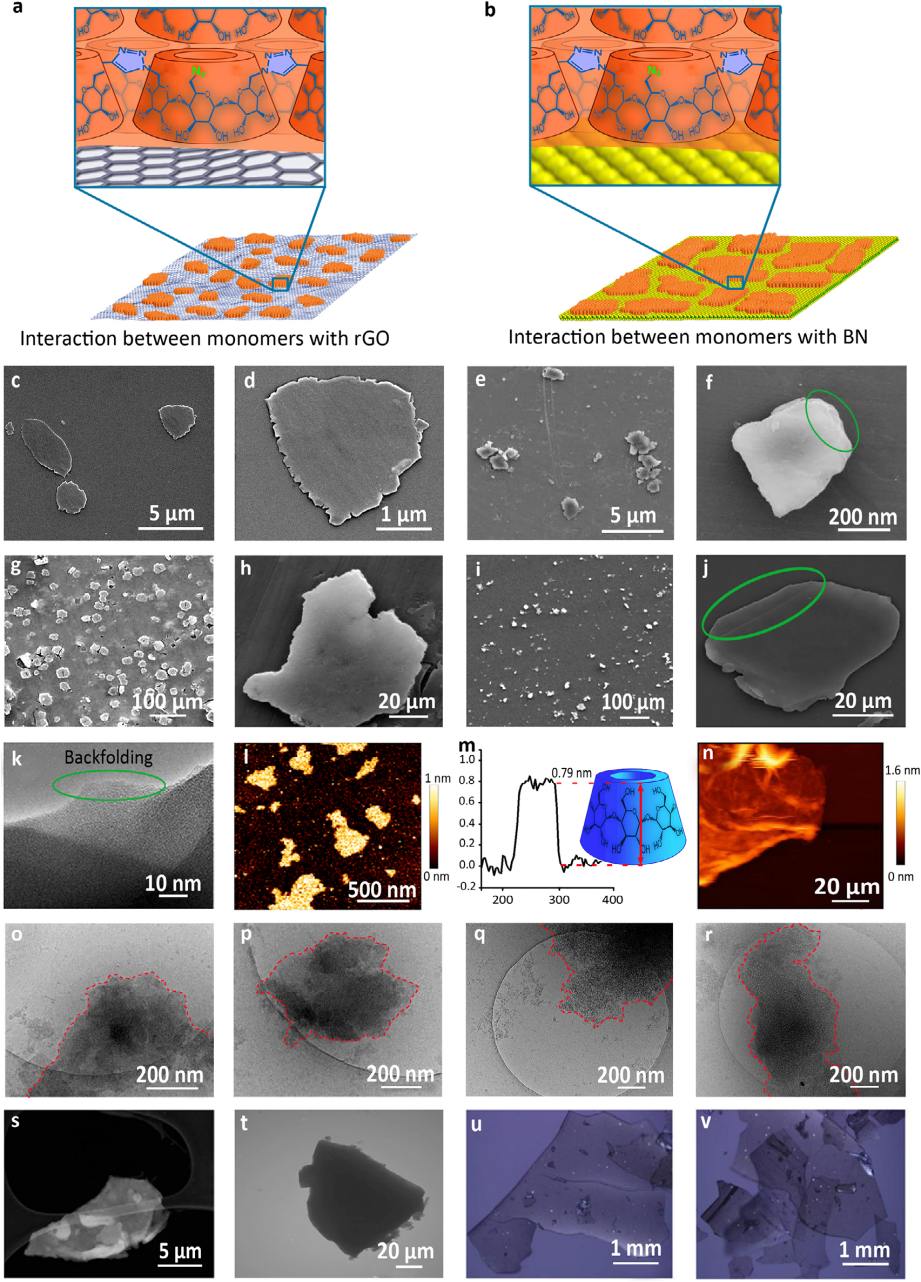
While strong interactions between monomers and rGO resulted in small sheets (a), the weaker interactions with BN and higher mobility of monomers led to 2D poly(*β*‐cyclodextrin)s with larger lateral sizes (b). SEM images of 2D‐CDs synthesized using rGO template (c,d) and their 2D‐CDSs counterparts (e,f). SEM images of 2D‐CDs synthesized using BN template (g,h) and their 2D‐CDSs counterparts (i,j). HR‐TEM image of 2D‐CDs synthesized using BN template (k). SFM image of 2D‐CDSs synthesized by rGO template (l) and its height profile (m) and 2D‐CDSs synthesized using BN template (n). Cryo‐TEM image of 2D‐CDs (o) and 2D‐CDSs (p) synthesized using rGO template. Cryo‐TEM image of 2D‐CDs (q) and 2D‐CDSs (r) synthesized using BN template. TEM images of 2D‐CDs (s) and 2D‐CDSs (t) synthesized using BN template. Optical microscopy images of 2D‐CDs (u) and 2D‐CDSs (v) synthesized using BN template.

Templates were dispersible in DMF, thus it was selected as the next solvent for 2D polymerization of monomers. Total energy interactions between rGO and BN templates and *β*‐CD‐N_3_ in DMF were in the range of −210.9 and −157.6 kJ mol^−1^, respectively (Figure 1c). However, relatively small particles with lateral sizes ≈ 100 nm were obtained, upon 2D polymerization (Figure 1c). This result showed that interactions between monomers and templates in DMF are strong enough to inhibit their mobility for a long‐range polymerization. Thus, their lateral crosslinking was limited to oligomers (Figure 1c; Movie S1, Supporting Information). To minimize interactions between the templates and monomers, water was added to DMF and the mixture was used as a reaction solvent. Interactions between templates and monomers in DMF/water mixture were weaker than each solvent individually (Figure 1d). This mixture was able to disperse templates and allows mobility of monomers on their surface. Microscopy images showed that long‐range lateral crosslinking of monomers on the surface of templates in DMF/water has successfully occurred. While 2D polymers with few hundred nanometers lateral size and ≈0.8 nm thickness was obtained using rGO, BN template resulted in several millimeters sheet‐like structures (**Figure** **2**). Simulation studies demonstrated that noncovalent interactions between monomers and rGO were ≈50 KJ mol^−1^ stronger than such interactions with BN. Stronger interactions between monomers and rGO restricted their mobility and limited their lateral crosslinking to small sheets (Figure 2a,c–f,l–p). Additionally, structural defects, pores, and oxygen‐containing functional groups within rGO basal plane must be taken into account as further template limitations. In contrast, the higher mobility of the monomers on BN facilitated their long‐range lateral crosslinking (Figure 2b). This was manifested in 2D‐CDs with large dimensions in the range of several micrometers to millimeters (Figure 2g–j,q–v). The stacked layers of 2D‐CDs can be observed in Figure 2f,j.

Supramolecular interactions between monomers and template should not be too strong to prevent mobility and reorganization of monomers and oligomers during polymerization. Dynamic interactions allow propagating sheets to heal some of the defects and cracks and extend polymerization to millimeter range.

On the other side, template/monomer interactions should be strong enough to organize monomers in a supramolecular 2D structure and inhibit leaking in the reaction environment. Such a narrow window is the key parameter dominating topology and physicochemical properties of 2D polymers. TEM images represented sheet‐like structures for 2D‐CDs, synthesized on BN template. Sheets were transparent and their underneath grid can be clearly seen (Figure 2s). TEM images of their sulfated counterparts, sheet‐like structures with clear edges and several micrometers lateral sizes (Figure 2t).

Moreover, HRTEM images of these sheets showed a flat amorphous/disordered structure without detectable unit cell (Figure 2k).

SFM images demonstrated 2D structures with lateral dimensions of 300–500 nm and height of 0.79 nm for 2D‐CDSs synthesized on rGO (Figure 2l,m). The height of such structures was corresponding to the height of a cyclodextrin unit.

Also, SFM images of 2D‐CDSs, synthesized on BN template, displayed very large sheets often with more than 100 micrometers lateral size (Figure 2n). Cryo‐TEM images of 2D‐CDs and 2D‐CDS, synthesized on BN (Figure 2o,p) and rGO (Figure 2q,r) template, showed 2D topology localized in the hole of the grid, indicating their hydrophilicity and integrated structures in aqueous mediums.

The secondary hydroxyl functional groups of 2D poly(*β*‐cyclodextrin)s (2D‐CDs) were changed to sulfate groups to boost their dispersibility in aqueous solutions and electrostatic interactions at biointerfaces (Figure 1a). After sulfation, the surface charge of 2D poly(*β*‐cyclodextrin)s turned to negative (‐44 mV) and their dispersibility in water increased (Figure S4A, Supporting Information).

**Figure 3:**
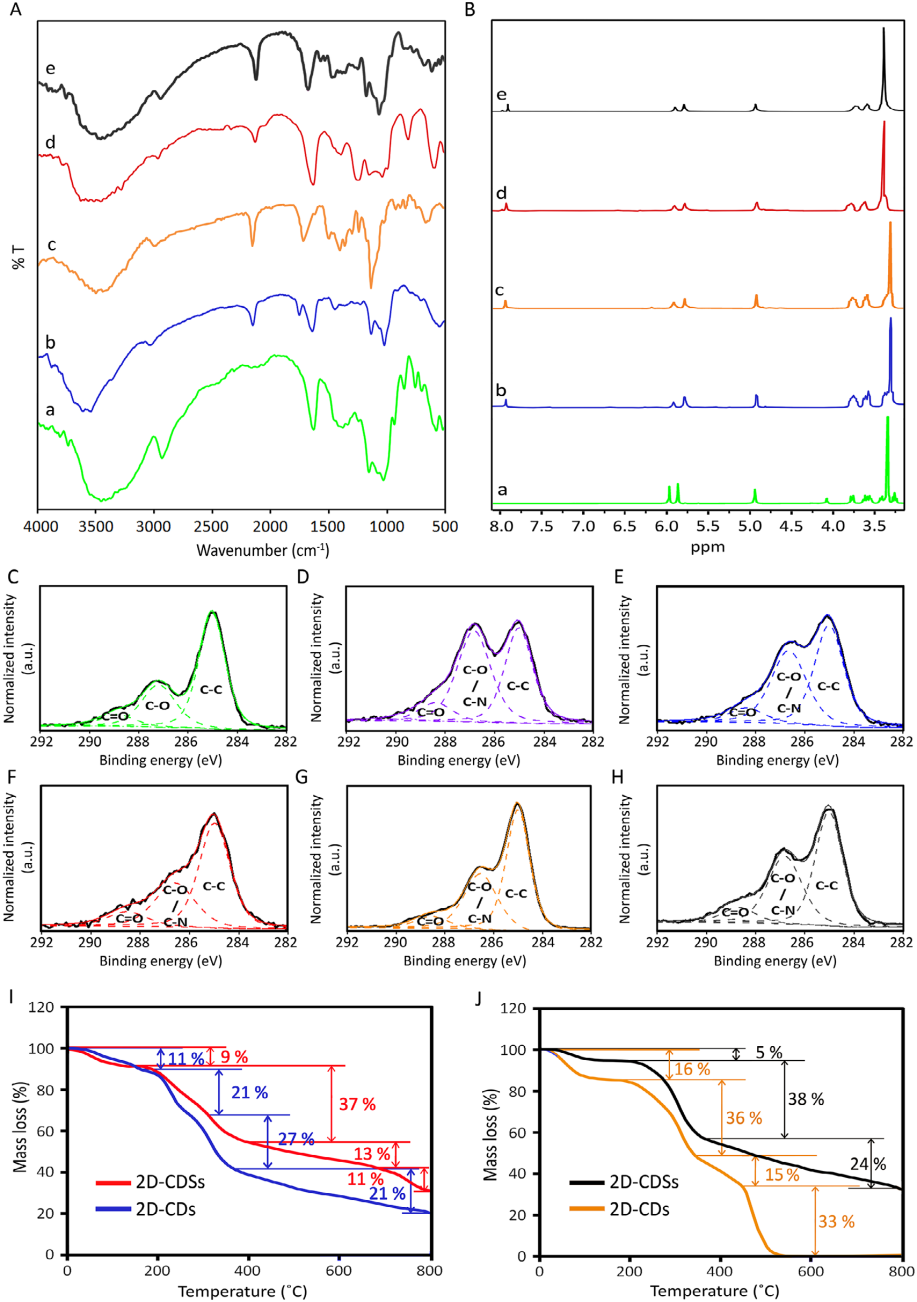
A) IR spectra of (a) *β*‐cyclodextrin), (b) 2D‐CDs synthesized on BN template, (c) 2D‐CDs synthesized on rGO template, (d) 2D‐CDSs synthesized on BN template, (e) 2D‐CDSs synthesized on rGO template. B) ^1^H NMR spectra of (a) *β*‐cyclodextrin), (b) 2D‐CDs synthesized on BN template, (c) 2D‐CDs synthesized on rGO template, (d) 2D‐CDSs synthesized on BN template, (e) 2D‐CDSs synthesized on rGO template. Highly‐resolved C1s XP spectra of C) *β*‐cyclodextrin, D) heptakis‐(6‐azido‐6‐deoxy)‐*β*‐cyclodextrin, E) and F) 2D‐CDs and 2D‐CDSs synthesized on BN template respectively, G) and H) 2D‐CDs and 2D‐CDSs synthesized on rGO template respectively. TGA thermograms of 2D‐CDs and 2D‐CDSs synthesized on BN I) and rGO J) templates, respectively.

The structure of 2D‐CD and their sulfated analogs (2D‐CDSs) were characterized using different spectroscopy methods. Weakening of the absorbance band of azide groups at 2100 cm^−1^, after click reaction, in comparison to heptakis‐(6‐azido‐6‐deoxy)‐*β*‐cyclodextrin (Figure S4Bc, Supporting Information) indicated efficient crosslinking of cyclodextrins on both templates (**Figure** **3**A). The appearance of an absorbance band at 1220 cm^−1^, which belongs to the stretching vibration of S = O bonds, was counted for the successful sulfation of the secondary functional groups of 2D‐CDs and production of 2D‐CDSs (Figure 3A).

Moreover, the weak absorbance band at 2100 cm^−1^, related to the unreacted azide functional groups, provide further possibilities for post‐modification of the smaller rim of 2D‐CDs. This is a route for the production of Janus 2D‐CDs, which is an ongoing project in our lab.

As shown in Figure 1a, Heptakis (6‐deoxy‐6‐azido)‐b‐cyclodextrin ((N_3_)_7_‐*β*‐CD) was synthesized according to previously reported methods in literature. Based on the ^1^HNMR spectrum (Figure S1, Supporting Information), selective replacement of all the primary hydroxyl groups of β‐CD with iodine atoms led to the formation of (I)_7_‐ *β*‐CD.

Also, (N_3_)_7_‐*β*‐CD was synthesized via reaction between NaN_3_ and (I)_7_‐ *β*‐CD. The ^1^H NMR spectrum of (N_3_)_7_‐*β*‐CD showed a complete disappearance of the primary hydroxyl proton signal at 4.5 ppm. This indicated the substitution of iodine atoms by azide groups and successful synthesis of (N_3_)_7_‐*β*‐C. 2D polymerization of *β*‐CD‐N_3_ by click reaction was also monitored by ^1^H NMR. After lateral crosslinking of monomers, 2D polymers were separated from templates and their ^1^H NMR spectra were recorded (Figure 3B). A signal at 7.95 ppm corresponded to the proton of the triazole ring, which was formed by click reaction between azide functional groups of *β*‐CD‐N_3_ and tripropargylamine as a crosslinker. Also, broadening of proton signals at 3.25–3.75 ppm was assigned to the polymerization of cyclodextrin monomers and successful synthesis of 2D‐CDs.

The synthesis of 2D polymers and their functionalization were also investigated by X‐ray photoelectron spectroscopy (XP). Based on survey XP spectra, *β*‐cyclodextrin was composed of carbon and oxygen, while the 2D‐CDs were mainly composed of carbon, oxygen and nitrogen, as expected (Figure S4Ca,Cc, Supporting Information).

Two components in the highly‐resolved C1s XP spectrum of *β*‐cyclodextrin at 285 and 287.2 eV were corresponding to the carbon atoms of C‐C and C‐O‐C bonds, respectively^[^ ^43^, ^48^, ^49^ ^]^ (Figure 3C). The increase in the intensity of the peak component at 286.8 eV, after the azidation of the primary hydroxyl groups of cyclodextrins was assigned to the newly formed C‐N bonds (Figure 3D). Moreover, a significant increase in the nitrogen content (5%) and intensity of N1s in the survey spectrum of *β*‐CD‐N_3_ was assigned to the successful azidation of *β*‐cyclodextrin (Figure S4Cb, Supporting Information).

After polymerization of *β*‐CD‐N_3_ on the rGO surface, the relative intensity of peaks at 286.7 eV and 288.4 eV decreased, because the peak component at 285 eV was significantly enhanced by carbon atoms of tripropargylamine (Figure 3E). The sulfation of secondary hydroxyl functional groups of 2D‐CDSs was manifested in the intensity of its peak components at 286.8 eV, owing to the formation of C─O─S bonds (Figure 3F).^[^ ^34^ ^]^ Also, survey and highly‐resolved S2p XP spectra showed a peak at around 170 eV that was corresponded to O─S bonds, confirming successful sulfation of 2D‐CDs synthesized on rGO template (Figure S4Db,Dc, Supporting Information). The same results were observed for 2D‐CDs and 2D‐CDSs synthesized on BN template (Figure S4Cc,Da, Supporting Information). In the highly‐resolved C1s spectrum of 2D‐CDs, synthesized on the BN template, the more intense peak component at 286.7 eV (49% of relative area) in comparison with its counterpart synthesized on rGO template (57% of relative area) was assigned to the lower number of tripropargylamine crosslinkers in its structure (Figure 3G). This result showed a lower degree of crosslinking in 2D‐CDs synthesized on BN in comparison with its counterpart synthesized on rGO template. We observed similar results for 2D‐CDSs synthesized on BN, where increased intensity of peak components at 286.8 eV and 288.7 eV indicated the presence of sulfate groups in this compound (Figure 3H). This result was confirmed by XRD diffractograms of the synthesized 2D‐CDs. 2D‐CDs synthesized using the rGO template showed two peaks at 2θ = 13° and 20° on broad background, indicating an amorphous structure with some crystalline domains. XRD diffractograms of 2D‐CDs synthesized on BN template showed a broad peak around 2θ 20°, which indicated an amorphous structure for this compound^[^ ^50^, ^51^, ^52^, ^53^ ^]^ (Figure S5C, Supporting Information). Sharper peaks in the diffractograms of polymers synthesized on rGO indicated a more crystalline structure than those synthesized on the BN template.

The synthesized 2D poly(*β*‐cyclodextrin)s showed different thermal properties compared to their precursors (Figure 3I,J; Figure S5A,B, Supporting Information). 2D‐CDs were mainly decomposed at 250–350 °C, owing to the decomposition of main cyclodextrin backbone (Figure 3I,J). Weight losses below 100 °C, in thermograms of 2D‐CDs and 2D‐CDSs, corresponded to the evaporation of water trapped in the cavity of cyclodextrins (Figure S5A, Supporting Information).^[^ ^54^ ^]^ The weight loss at 100–220 °C corresponded to the detachment of unreacted azide functional groups as nitrogen gas (Figure S5B, Supporting Information). Sulfate groups change the thermal stability significantly. Similar thermal behavior for the sulfated polymers is reported in the literature.^[^ ^55^ ^]^

After characterization of 2D poly(*β*‐cyclodextrin)s, their toxicity against A549 and HBE cell lines using CCK8 assay was evaluated. 2D‐CDSs showed higher biocompatibility than 2D‐CDs, because of higher dispersibility in aqueous medium and negative surface charge. It didn't show a significant toxicity up to 0.5 mg mL^−1^ against both cell lines (Figure S6, Supporting Information).

*β*‐cyclodextrin and its derivatives are able to from inclusion complexes with biomolecules such as cholesterol and extract it from biological mediums and cell compartments.^[^ ^56^, ^57^, ^58^ ^]^ Based on this fact, loading capacity of 2D‐CDSs to load and deliver cholesterol was studied. HPLC and UV measurements showed a loading capacity of 0.66 mg mg^−1^ for cholesterol, which shows a higher loading capacity compared to other β‐cyclodextrin‐based polymers^[^ ^59^, ^60^, ^61^ ^]^ (Figure S7a, Supporting Information).

Previous studies have demonstrated that β‐cyclodextrin derivatives possess a remarkable capacity to encapsulate hydrophobic molecules such as cholesterol. However, the accessibility of their cavities can be restricted by the conformation of the supporting platforms or polymer backbone. 2D‐CDSs, due to the large number of cyclodextrins and accessible surface area, are able to host the guest molecules efficiently, leading to a higher loading capacity. The multivalent host‐guest interactions between 2D poly(*β*‐cyclodextrin)s and biosystems containing cholesterols should give rise to interesting biological behaviors that are important in nanomedicine.

We selected 2D‐CDs and 2D‐CDSs synthesized on rGO template for interactions at biointerfaces, because of their smaller lateral size and higher dispersibility in aqueous solutions.

**Figure 4:**
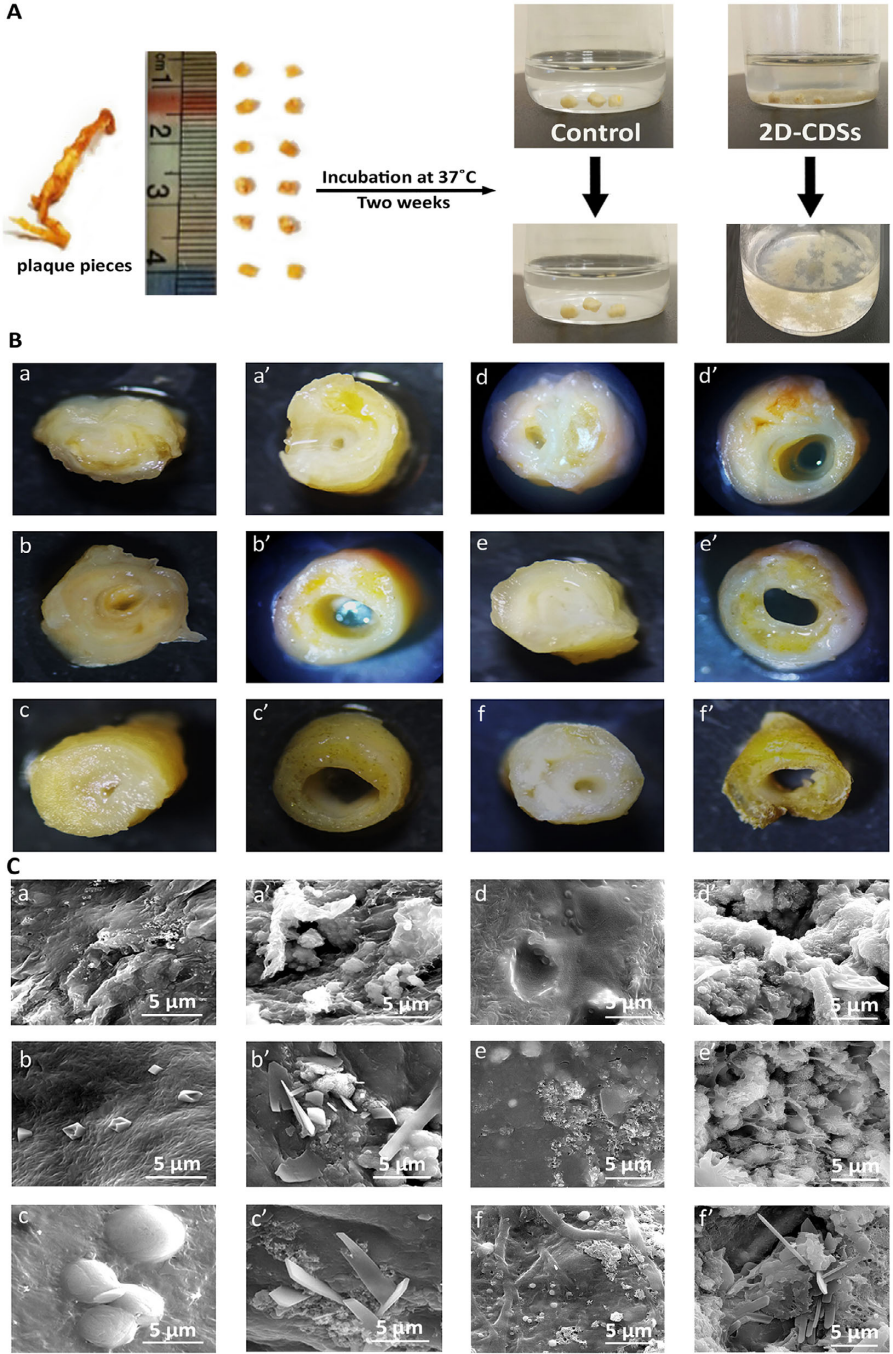
A) An atherosclerosis plaque divided into small pieces and used for interaction with 2D‐CDSs. Pieces incubated in PBS were used as control samples. Optical microscopy B) and SEM C) images of plaques pieces in the absence (a, b, c) and presence (a’, b’, c’) of 2D‐CDs as well as in the absence (d, e, f) and presence (d’, e’, f’) of 2D‐CDSs after 1, 3 and 14 days, respectively.

It is known that cholesterol is one of the main components of vascular plaques. To investigate the ability of the synthesized 2D‐CDs for multivalent interactions with plagues and extracting cholesterol from their structures, plaques were collected from patients after surgery, divided into several pieces and incubated with 2D‐CDs for two weeks. The morphology and composition of the plaques were analyzed and compared with control experiments (**Figure** **4**; Figure S7b, Supporting Information).

Optical microscopy images showed a clear difference between plaque species incubated with 2D‐CDs and the control experiments. The plaque wall became thin after three days of incubation with 2D‐CDs and 2D‐CDSs and broken into small pieces within two weeks (Figure 4A–C; Figure S7b, Supporting Information).

Corrosion and changes in the structure of plaques were detected by SEM after 3 days (Figure S7c, Supporting Information). The smooth surface of plaques was converted into a rough and porous structure upon incubation with 2D‐CDs and 2D‐CDSs. From optical microscopy and SEM images, it can be concluded that both 2D‐CDs and 2D‐CDSs are able to efficiently disrupt plaques (Figure 4B,C). This is attributed to the multivalent host‐guest interactions between cholesterol moieties in the structure of plaques and cavities of 2D‐CDs.

To investigate this assumption, the compositions of the plaques, were monitored at different times points after incubation with 2D‐CDSs. In the first three days, the control plaques and those treated with 2D‐CDSs showed similar compositions.

The carbon and nitrogen contents of plaques were about 50 wt.% and 18 wt.% respectively and their calcium and phosphorous contents were below 2 wt.% (Figure S8a, Supporting Information). After two weeks, the calcium and phosphorous contents of control plaques increased to 10–12 wt.%, while that of the plaques incubated with 2D‐CDS decreased to <2 wt.% (Figure S8b, Supporting Information).

**Figure 5:**
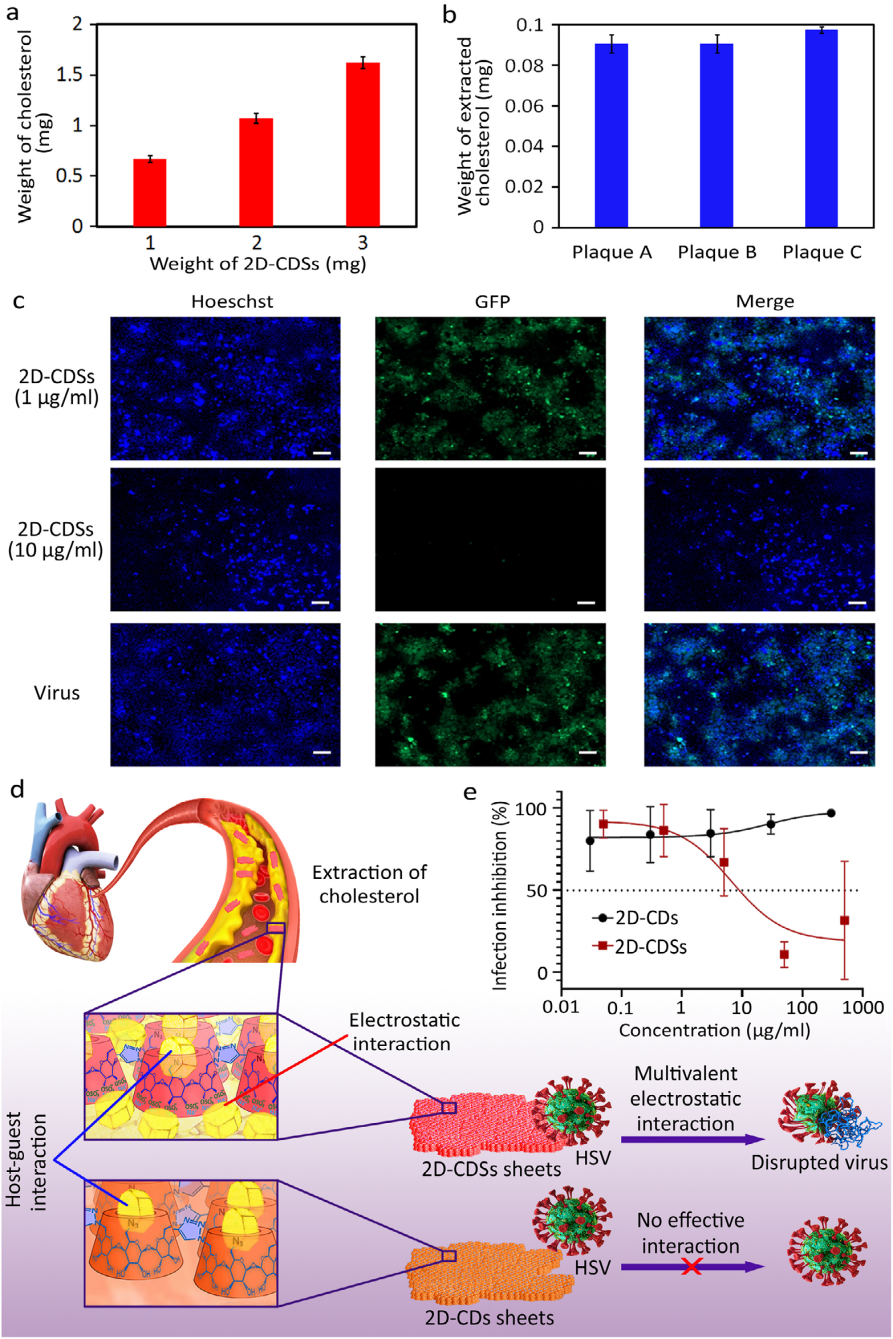
a) The ability of different concentrations of 2D‐CDSs (1, 3, and 5 mg mL^−1^) to load cholesterol (5 mg mL^−1^). b) The amount of cholesterol extracted from 1 mg of plaques by 1 mg of 2D‐CDSs. Plaques were collected from three different patients so called A, B and C. c) Immunofluorescent images for the HSV‐infected cells in the presence of 1 and 10 µg mL^−1^ concentrations of 2D‐CDSs. While in the absence of 2D‐CDSs (bottom row) or at low concentrations of this compound (upper row) significant infection is observed, higher concentration (10 µg mL^−1^) of this compound inhibited infection of cells by HSV significantly (Scale bar: 100 µm). d) Sulfation of 2D‐CDs improves their interactions at biointerfaces, due to the multivalent electrostatic forces. They can interact with plaques by synergistic electrostatic and host‐guest interactions and incapacitate viruses by electrostatic interaction as the main driving force. e) Plaque reduction ratios for the 2D‐CDSs at different concentrations. While 2D‐CDs did not significantly reduce plaques, their sulfated counterparts had an IC50 of 1.14 nM. Scale bar: 100µm.

To understand the dramatic changes in the calcium and phosphorous contents of the plaques in the control experiments, EDX of the surface and cross‐section of plaques were recorded (Figure S8b, Supporting Information). We found different compositions for different plaques layers. The calcium and phosphorous contents of plaques increased from the surface to the inner section. Based on this result, we assumed that a thin layer of plaque surface, mainly carbon‐containing organic materials, was washed away upon incubation with PBS and the main composition with high calcium and phosphorous content was revealed after several days. The low calcium and phosphorous contents of 2D‐CDSs treated plaques, after two weeks, indicated the high ability of this compound to extract these elements from plaques. This was assigned to the coordination of sulfate groups of polymers to calcium and calcium ions containing phosphate groups.^[^ ^62^, ^63^, ^64^ ^]^ The ability of sulfate groups to bind calcium ions in biological systems is well documented in literature.^[^ ^62^, ^63^, ^64^ ^]^ Moreover, removing phosphate‐containing species disrupts the crystalline structure of plaques, rendering them more penetrable to 2D‐CDSs. The efficient penetration of 2D‐CDSs, combined with the exclusion of cholesterol through host–guest interactions, accelerates plaque degradation in a shorter time.^[^ ^65^, ^66^ ^]^ PBS solution of 2D‐CDSs (5 mg mL^−1^) was added daily to a plaque piece in PBS and the surface and composition of the plaque were analyzed. In control experiments (absence of 2D‐CDSs), plaque pieces were incubated only with PBS. Removing the calcium and phosphate ions along with extraction of cholesterol was the main mechanism of plaque destruction by 2D‐CDSs. The separation of cholesterol from plaque by 2D‐CDSs was investigated using HPLC. 2D‐CDSs were incubated with plaques in PBS for two weeks and the supernatant was injected into the HPLC. To accurately determine the retention time of cholesterol, the standard sample (10 µl, 5 ppm) was added to the plaque and an increase in the intensity of the cholesterol peak at 2.9 min was counted as a contribution (Figure S9, Supporting Information). The ability of different concentrations of 2D‐CDSs namely 1, 3, and 5 mg mL^−1^ to absorb cholesterol from a 5 mg mL^−1^ solution was 0.66, 1.07, and 1.62 mg, respectively (**Figure** **5**a). The cholesterol contents of the supernatant of treated plaques with 2D‐CDSs solution (1 mg mL^−1^) were evaluated. It was found that the polymer is able to extract ≈9 × 10^−2^ mg cholesterol from 1 mg of plaques (Figure 5b). The results for all three different plaque samples were almost the same. This may be due to the saturation and maximum amount of cholesterol that 2D‐CDSs can separate from plaques. Considering the amount of cholesterol hosted in the cavities of 2D‐CDs together with this excess amount in the supernatant, 2D‐CDSs showed a high ability to extract cholesterol from plaques.

This can be the main reason for destruction plaque crystalline structure after incubation with 2D‐CDs. The high loading capacity and strong multivalent host‐guest and electrostatic interactions at the plaques/2D‐CDSs interface indicated the high potential of 2D cyclodextrins for various biomedical applications, including the treatment of atherosclerosis, drug delivery and virus inactivation. Previously, we have shown that 2D materials bearing negatively charged functional groups are able to bind viruses via electrostatic interactions.^[^ ^55^, ^67^, ^68^ ^]^

The high surface area, accessibility of functional groups and flexibility are important factors that boost their ability to capture and inactivate viruses. For example, 2D polyglycerols with the negative surface charge have shown stronger virus inhibition compared to similar 3D nanomaterials.^[^ ^34^ ^]^

Moreover, the combination of different driving forces in one system results in high virucidal activity, as demonstrated by the synergistic effect of hydrophobic and electrostatic interactions.^[^ ^69^, ^70^ ^]^ Accordingly, 2D‐CDs with the ability of multivalent interactions at biointerfaces were investigated for interactions with HSV and inhibit the infection of VeroE6 cells.

Herein, the infected cells were marked green by green fluorescent protein (GFP) and the total cells were marked blue by cell nuclei staining.

While 2D‐CDs didn't show significant interaction with HSV, their sulfate counterparts efficiently inhibited infection (Figure 5c). The inertness of 2D‐CDs against HSV, suggested that interaction between the polymer and virus is triggered by electrostatic forces. Our data, regarding interactions between 2D‐CDs and bio‐objects including plaque and HSV indicated a crucial role for sulfate groups in multivalent interactions at these biointerfaces (Figure 5d). The IC50 of 2D‐CDSs for inhibition HSV was 6.6 µg mL^−1^, which corresponds to ≈1.14 nM considering the molecular weight of 2D‐CDSs (Figure 5e) (for calculation of the molecular weight of 2D‐CDSs see ESI).

## 3 Conclusion

2D‐CDs were synthesized by lateral crosslinking of cyclodextrin monomers on two types of colloidal templates. The noncovalent interactions between cyclodextrin monomers and templates were the main driving forces for 2D polymerization and dominated the lateral size of 2D‐CDs. These driving forces were manipulated by changing the solvent and optimized to obtain flat sheets of CDs. The type of template was another factor that affected the lateral size of 2D‐CDs dramatically. Stronger interactions between the template and monomers restricted their mobility and led to smaller sheets.

Due to their ability for multivalent host‐guest interactions at biointerfaces, carbohydrate backbone and controlled functionalization of both sides, 2D‐CDs are promising candidates for a wide range of future biomedical applications, such as the inhibition of viruses and the destruction of vascular plaques.

## 4 Experimental Section

Details regarding materials, methods and synthesis and characterizations can be found in ESI.

## Conflict of Interest

The authors declare no conflict of interest.

## Supporting information



Supporting Information

Supplemental Movie 1

## Data Availability

The data that support the findings of this study are available in the supplementary material of this article.
