# Supplementary material for: Two‐Dimensional Polycyclodextrins for Strong Multivalent Host‐Guest Interactions at Biointerfaces
Source: Small. 2025 Apr 27;21(23):2412282. doi: 10.1002/smll.202412282 (PMC12160674; doi:10.1002/smll.202412282)
Supplement: Supplementary file 1 — Supporting Information [file SMLL-21-2412282-s001.docx]

Supporting Information

Two-Dimensional Polycyclodextrins for Strong Multivalent Host-Guest Interactions at Biointerfaces

*Zahra Goudarzi^1#^, Zahra Mohammadi^1#^, Reza Maleki^2^, Siamak Beyranvand^1*^, Chuanxiong Nie^3^, Mohammad Fardin Gholami^4^, Özge Akkaya^3^, Mahdieh Kalantari^1^, Mohammad Nemati^1^, Fatemeh Yousufvand^1^, Fatemeh Shahverdi^1^, Marzieh Rashidipour^5^, Zeinab Ahmadian^6^, Ievgen Donskyi^3,7^, Philip Nickl^3,7^, Marek Brzeziński^8^, Kai Ludwig^9^, Jürgen P. Rabe^4^, Raul Arenal^10,11,12^, Cheng Chong^13^, Angelo H. ALL^14^*, Mohsen Adeli^1,3*^*

^1^Department of Chemistry, Lorestan University, Khorramabad, Iran

^2^Department of Chemical Technologies, Iranian Research Organization for Science and Technology (IROST), Tehran, Iran

^3^Institut für Chemie und Biochemie, Freie Universität Berlin, Takustr. 3, 14195 Berlin, Germany

^4^Department of Physics & IRIS Adlershof, Humboldt-Universität zu Berlin, Newtonstrasse 15, 12489 Berlin Germany

^5^Environmental Health Research Center, Lorestan University of Medical Sciences, Khorramabad, Iran

^6^Department of Pharmaceutics, School of Pharmacy, Lorestan University of Medical Sciences, Khorramabad, Iran

^7^BAM – Federal Institute for Material Science and Testing, Division of Surface Analysis and Interfacial Chemistry, Unter den Eichen 44-46, 12205 Berlin, Germany

^8^Division of Polymers, Centre of Molecular and Macromolecular Studies, Polish Academy of Sciences, Sienkiewicza 112, 90-363 Łódź

^9^Forschungszentrum für Elektronenmikroskopie and Core Facility BioSupraMol, Institut für Chemie und Biochemie, Freie Universität Berlin, Fabeckstr. 36a, 14195 Berlin, Germany

^10^Instituto de Ciencias de Materiales de Aragon (INMA), CSIC-Universidad de Zaragoza, 50009 Zaragoza, Spain

^11^Laboratorio de Microscopias Avanzadas (LMA), Universidad de Zaragoza, 50018 Zaragoza, Spain

^12^Fundacion ARAID, 50018 Zaragoza, Spain

^13^College of Polymer Science and Engineering, State Key Laboratory of Polymer Materials Engineering, Sichuan University, Chengdu, 610065, China

^14^Department of Chemistry, Hong Kong Baptist University, Hong Kong SAR, China

Keywords: Two-dimensional polymers, Polycyclodextrins, Two-dimensional carbohydrates, Multivalent host-guest interactions, Antiviral.

# Zahra Goudarzi^1#^, Zahra Mohammadi^1#^ contributed equally.

Keywords: Two-dimensional polymers, Polycyclodextrins, Multivalent host-guest, Virus inhibition, Atherosclerosis.

*Corresponding Authors

E-mail: m.aadeli@fu-berlin.de

E-mail: sb1984sb2@gmail.com

[Experimental 4](#_Toc193469699)

[Materials 4](#_Toc193469700)

[Methods 5](#_Toc193469701)

[Synthesis of heptakis (6-deoxy-6-iodo)-b-cyclodextrin (I_7_-β-CD) 15](#_Toc193469702)

[Synthesis of heptakis (6-azido-6-deoxy)-*β*-cyclodextrin ((N_3_)_7_-β-CD) 15](#_Toc193469703)

[Synthesis of two-dimensional polycyclodextrins on colloidal templates via click chemistry (2D-CDs) 15](#_Toc193469704)

[Synthesis of sulfated two-dimensional polycyclodextrin (2D-CDSs) 16](#_Toc193469705)

[Interactions between 2D-CDs and atherosclerotic plaques 17](#_Toc193469706)

[Investigation of the loading capacity of 2D-CDs for cholesterol and their ability to suck out this compound from plaques by HPLC 17](#_Toc193469707)

[Determination of loading capacity: 18](#_Toc193469708)

[Sucking out cholesterol from plaques 18](#_Toc193469709)

[Characterizations 19](#_Toc193469710)

[Figure S1. ^1^H NMR spectra of (a) *β*-cyclodextrin, (b) heptakis-(6-deoxy-6-iodo-)-*β*-cyclodextrin, (c) heptakis-(6-azido-6-deoxy)-*β*-cyclodextrin. 19](#_Toc193469711)

[Figure S2. SEM images of (a, b) BN and (c, d) rGO that were used as templates for the synthesis of 2D-CDs. 20](#_Toc193469712)

[Self-assembly of heptakis (6-azido-6-deoxy)-*β*-cyclodextrin ((N_3_)_7_-β-CD) on highly oriented pyrolytic graphite 20](#_Toc193469713)

[Figure S3. (a and b) SFM-QI height image of the freshly cleaved HOPG surface acquired at ambient conditions (23 ^o^C and 32% relative humidity). (c) SFM-QI height image of (N_3_)_7_-*β*-CD) molecules at DMF-HOPG interface. (d) Close up view of the center of figure (c). (e) SFM-AC height image of the ((N_3_)_7_-*β*-CD) molecules at DMF-HOPG interface. (f) Close up of the self-assembled layers over the HOPG surface. (g) Demonstration of the self-assembled layer of ((N_3_)_7_-*β*-CD) over HOPG surface in DMF solvent and (h) the height distribution of the HOPG and (N_3_)_7_-*β*-CD) layer. (i) Schematic view of in solvent (liquid) SFM measurement of the self-assembly of (N_3_)_7_-β-CD over HOPG layered crystal. 23](#_Toc193469715)

[Figure S4. (A) Zeta potential diagram of (a) 2D-CDs and (b) 2D-CDSs. The surface charge of 2D-CDSs was slightly negative, due to the presence of high amount of nitrogen. After sulfation the negative surface charge increased to -44 mV. (B) IR spectra of precursors of 2D-CDs including (a) *β*-cyclodextrin, (b) heptakis (6-deoxy-6-iodo)-*β*-cyclodextrin (I_7_-*β*-CD) and (c) heptakis (6-azido-6-deoxy)-*β*-cyclodextrin (N_3_)_7_-*β*-CD. Based on XPS spectra, 2D-CDs polymers were mainly composed of carbon, oxygen and nitrogen, as it was expected. Survey XPS spectra of (C) (a) *β*-CD, (b) heptakis (6-azido-6-deoxy)-*β*-cyclodextrin (N_3_)_7_-*β*-CD, (c) 2D-CDs synthesized on BN templates. (D) (a)2D-CDSs synthesized on BN templates, (b) 2D-CDs and (c) 2D-CDSs synthesized on rGO templates respectively. Highly resolved S2p XPS spectra of 2D-CDSs synthesized on: (E) BN and (F) rGO templates. Highly resolved S2p XPS spectrum of 2D-CDSs synthesized on rGO template showed a doublet peak at 169.3 eV, confirming successful sulfation of 2D-CDs. Appearance of S2p peak at 168.9 eV after sulfation of 2D-CDs synthesized on BN template confirms successful production of 2D-CDSs. However, for both non-sulfated samples no sulfur is detected. 25](#_Toc193469716)

[Figure S5. TGA thermogram of (A) *β*-cyclodextrin, demonstrating a weight loss (14%) at 120 °C, due to the evaporation of water and a main weight loss (77%) at 350 °C, assigned to destruction of it backbone. (B) heptakis (6-azido-6-deoxy)-*β*-cyclodextrin ((N_3_)_7_-*β*-CD, demonstrating a main weight loss (51%) at 220 °C. Decreasing the thermal stability of cyclodextrin after azidation can be assigned to the decomposition of azide functional groups and releasing nitrogen gas. (C) Powder XRD diffractograms of (a) 2D-CDs and (b) 2D-CDSs synthesized on BN template respectively, (c) 2D-CDs and (d) 2D-CDSs synthesized on rGO template respectively. 26](#_Toc193469717)

[Toxicity of the synthesized materials 27](#_Toc193469718)

[Figure S6. The cytotoxicity of 2D-CDs and 2D-CDSs, synthesized on rGO template, against A549 (a) and HBE (b) cell lines using CCK8 assay. Toxicity of 2D-CDs was higher than that for 2D-CDSs and varied by changing the cell line. The higher toxicity of 2D-CDs can be assigned to its lower solubility and big agglomerations. 2D-CDSs showed a high biocompatibility against both cell lines and didn’t show significant toxicity up to 0.5 mg/ml. 27](#_Toc193469719)

[Loading and sucking out cholesterol from plaques by 2D-CDSs 28](#_Toc193469720)

[Figure S7. (a) Calibration curve of obtained using different concentrations of methanol solution of cholesterol in the range of 1-5 μg/ml. Evaluating of the (b) optical microscopy and (c) SEM images of pieces of plaques in the absence (i-iii) and presence (i’-iii’) of 2D-CDSs after 1, 3 and 14 days, clearly showed the ability of both materials to extract cholesterol from plaques. Plaques were broken down after two weeks interactions with 2D-CDSs. 29](#_Toc193469721)

[Figure S8. (a) Evaluation of the composition of plaques in the absence (control) and presence of 2D-CDSs after and one and 14^th^ days of incubation from the surface and cross section of plaques by EDX. (b) Composition of plaques in terms of calcium and phosphorus measured in the absence (control) and presence of 2D-CDSs after and one and 14^th^ days of incubation from the surface and cross section of plaques by EDX. 30](#_Toc193469722)

[The loading capacity was calculated by HPLC using this equation: 31](#_Toc193469723)

[Figure S9. (a) Evaluation of the area under the curve of stock concentration of cholesterol (1-5 µg/ml). Investigation of the host-guest interaction between (b) cholesterol (5 mg/ml) and (c) plaque (1 mg) different concentration of 2D-CDSs (1, 3 and 5 mg/ml) using HPLC. 31](#_Toc193469724)

[Estimation of the molecular weight of 2D-CDSs synthesized using graphene template 32](#_Toc193469725)

Movie S1..…………………………………………………………………………………….32

[References 32](#_Toc193469726)

# Experimental

# Materials

*β*-cyclodextrin (C_42_H_70_O_35_), tripropargylamine (C_9_H_9_N), triphenylphosphine (C_18_H_15_P), sodium azide (NaN_3_), boron nitride (BN), graphite, sodium methoxide (CH_3_ONa), pyridine sulfur trioxide (C_5_H_5_NSO_3_), sodium ascorbate (C_6_H_7_NaO_6_), copper sulphate (CuSO_4_), dimethylformamide (CH_3_H_7_NO), methanol (CH_3_OH) were purchased from Merck (Germany) company and Sigma-Aldrich (Schnelldorf, Germany). Biotech cellulose ester dialysis bag (MWCO=2 kDa) was purchased from Spectra/Por.

Plaque samples (right coronary endarterectomy) related to patients: a 51 years old woman with high blood pressure diabetes risk factor disease, a 63 years old woman with high blood pressure, diabetes, passing lipidemia and a 50 years old man with high blood pressure were collected post-surgery in Tehran Heart Center Hospital and approved by the research ethics committee. The ethic code for the plaques received form patients is IR.LUMS.REC.1403.339.

# Methods

Fourier transform infrared (FTIR) spectra were recorded by Bruker-Tensor 320 FTIR Spectrometer at a scanning range from 4000 cm^-1^ to 400 cm^-1^. Elemental analysis was carried out using ECS 4010 instrument (NC technologies (Costech)) with four columns and detector for C, H, S and N elements. The morphology and surface structure of materials were investigated using LEO440I electron scanning microscopy (SEM) under vacuum at an operating voltage of 10 kV. Before analysis, all samples were sprayed with gold by sputtering for 15 s. SEM was equipped with an X-ray energy dispersion microanalysis system (Energy dispersive X-ray) (EDX) that was able to identify the number of relevant elements in the compounds. X-ray diffractograms (XRD) were recorded using Halland Philips Xpart instrument (Cuk, radiation, λ=0.154056 NM) in the range of 2Ө = 10-80˚ and with a scanning rate of 2˚/Min. Thermogravimetric analysis (TGA) was recorded by STA PT1600 Linseis(Robbinsville,USA) ,(Netzsch TG 209 f1 lris) under nitrogen at range 25 °C to 800 °C range with 10 °C /min heating rate. ^1^H NMR and ^13^C NMR spectra were recorded on a Jeol ECX 400 spectrometer or on a Bruker BioSpin Avance 700 spectrometer (Bruker Corporation, Billerica, MA, USA) (at 295 K). Chemical shifts were reported in ppm using the deuterated solvent peak as the internal standard and tetramethyl silane (SiMe_4_) was used for internal calibration at 125 MHz with complete proton decoupling.

X-ray photoelectron spectroscopy (XPS), for a correct visualization of the 2D polymers by cryo-TEM was performed for analysis of cyclodextrin derivatives samples deposited thereon were cleaned in piranha solution (1:4) 30% H_2_O_2_: 98% H_2_SO_4_ (v/v) during ultrasonication at room temperature for 10 min. Then they were washed with the DI water 5 times and with acetone 2 times. After drying overnight, the studied compounds were dissolved in methanol and evenly distributed dropwise across the surface of gold substrates. XPS spectra were recorded using a Kratos Axis Ultra DLD spectrometer equipped with a monochromatized Al Kα X-ray source (1486.69 eV) using an analyzer pass energy of 80 eV for survey spectra that were used for quantification. High-resolution, core-level O1s, C1s, S2p, and N1s spectra were recorded in FAT (fixed analyzer transmission) mode at a pass energy of 20 eV. Both the electron emission angle and the source-to-analyzer angle were 60°. The binding energy scale of the instrument was calibrated following a Kratos Analytical Ltd procedure that used ISO 15472 binding energy data. Spectra were recorded by setting the instrument to the hybrid lens mode and the slot mode, which provided approximately a 300 x 700 µm^2^ analysis area and using charge neutralization. All XPS spectra were processed with the UNIFIT program (version 2022). A Gaussian/Lorentzian product function peak shape model GL (30) was used in combination with a Shirley background. If not otherwise denoted, the L-G mixing for component peaks in all spectra were constrained to the value of 0.37. Peak fitting of C1s spectra was performed by using a symmetric peak shape model for all component peaks. After peak ﬁtting of the C1s spectra, all the binding energies were calibrated in reference to the aliphatic C1s component at a binding energy of 285.0 eV.

Some of the HRTEM images have been obtained using a Cs-corrected Thermo Fisher Scientific Titan-Cube microscope working at 80 kV. In addition, some scanning TEM images were acquiring a probe-corrected Thermo Fisher Scientific Titan Low Base microscope operated also at 80 kV.

For a correct visualization of the 2D polymers was used Cryo-Transmission electron microscopy (cryo-TEM).Microscopic 200-mesh grids covered with a perforated carbon film (R1/4 batch of Quantifoil, MicroTools GmbH, Jena, Germany) were cleaned with chloroform and hydrophilized by 60 s glow discharging at 10 µA in a EMSCOPE SC500. Then 4 µl aliquots of the corresponding sample solution were applied to the grids. The samples were vitrified by automatic blotting and plunge freezing using a FEI Vitrobot Mark IV (Thermo Fisher Scientific Inc., Waltham, Massachusetts, USA) using liquid ethane as cryogen. The vitrified specimens were transferred to the autoloader of a FEI TALOS ARCTICA electron microscope (Thermo Fisher Scientific Inc., Waltham, Massachusetts, USA). This microscope is equipped with a high-brightness field-emission gun (XFEG) operated at an acceleration voltage of 200 kV. Micrographs were acquired on a FEI Falcon 3 direct electron detector (Thermo Fisher Scientific Inc., Waltham, Massachusetts, USA) with a 100 µm objective aperture using the microscope's low-dose protocol.

Scanning force microscopy (SFM)

The optical images of 2D-CDs and morphology of plaques after treatment with the synthesized materials were investigated by optical microscopy, Nikon SMZ-745 Stereo Microscope. A Shimadzu HPLC device (SCL-10AVP) HPLC equipped with a C18 column (250×4.6 mm, 5 µm), UV–vis detector (SPDM10Avp), quaternary pump (LC-10ATvp), vacuum degasser and system controller (SCL10Avp), manual injector with a 10 µL sample loop was used to investigate the loading capacity of 2D-CD s for cholesterol and determination the ability of these materials to suck out cholesterol from Plaque samples. Shimadzu’s LabSolutions software was used for the process of chromatographic data. Identification of cholesterol was performed using mobile phase consisting of acetonitrile and isopropanol solvent, HPLC grade, with a ratio (v:v) of 30:70. The column oven, flow rate and UV detector were set at ambient temperature, 1.0 ml/min and 239 nm, respectively. All samples were repeated three times (n=3). Retention time (RT) for cholesterol according to the spiked sample was 2.9/min.

HSV-1 infection and inhibition

HSV-1-GFP was kindly provided by Dr. Jakob Trimpert and Prof. Dr. Nikolaus Osterrieder, Freie Universität Berlin. It was propagated in Vero E6 cells and titrated by plaque assay before usage.

For pre-infection inhibition assay, the Vero E6 cells were seeded into 96-well plates. After being washed with phosphate-buffered saline (PBS), the cells were incubated with samples of different concentrations for 45 min, followed by adding HSV-1-GFP solutions to reach a multiplicity of 0.1. After 24h incubation and fixation, Hoechst was used to stain cell nuclei. The images were acquired on a Zeiss Axio Observer Apotome microscope (ZEISS, Germany).

For plaque reduction assay, HSV (1×10^3^ PFU/mL) was pre-treated with samples of different concentrations and heparin, then inoculated on Vero E6 cells for virus binding at r.t. for 45min. Afterwards, the cells were washed with PBS. Then, the cells were cultured for 72 h with overlay medium for plaque formation. Finally, the plaque reduction ratios are calculated by comparing plaque forming units of the sample-treated virus solution and the non-treated virus solution.

Simulation studies

First, cyclodextrin (CD) molecules were constructed using Avogadro software. After the initial structure was created, these molecules were geometrically optimized using CP2K software and BLYP functional with D3 dispersion correction to accurately model the interatomic interactions. In this process, STO-3P basis set was used for all atoms. Also, structural optimization was performed under NVT ensemble at 300 K using Nosé-Hoover thermostat. After optimization, the electrostatic charges of CD molecules were calculated using ESP method using CP2K software. Then, these charges were parameterized through PolyParGen website to make CD molecules compatible with OPLS-AA force field for molecular dynamics simulation. Dimethylformamide (DMF) solvent was also prepared in a similar way. In this process, the molecular structure of DMF was first optimized using CP2K, then its ESP charges were calculated and parameterized via PolyParGen to match the OPLS-AA force field. To investigate the interactions of cyclodextrin with nanomaterials, each CD molecule was placed near a graphene (rGO) nanostructure and a boron nitride (BN) nanostructure, separately, in the presence of three different solvent environments: water, DMF, and a water/DMF mixture with a molar ratio of 1:1. At this stage, each system was placed inside a cubic simulation box with periodic boundary conditions, which was created using gmx editconf. Then, solvent addition was performed using the gmx solvate command. Molecular dynamics simulations were performed using GROMACS 5 software under the NPT ensemble at 300 K and 1 atm. A Parrinello-Rahman barostat was used to control the pressure and a Nosé-Hoover thermostat was used to control the temperature. Also, the particle mesh Ewald (PME) method was used to calculate electrostatic interactions and a 12 Å cutoff was used for van der Waals interactions. Each system was first equilibrated for 1 ns under the NVT ensemble and then a 1 ns simulation was run under the NPT ensemble. The stability of the systems was investigated through RMSD and RDF analyses and the interaction energies were analyzed to determine the preferential adsorption behavior of cyclodextrin on the two types of nanostructures in different solvent environments. To calculate the interaction energies between cyclodextrin and nanostructures, the MMPBSA method was used. This method calculates the interaction energies between the ligand and the nanostructure surface in the solvent environment and includes two parts: van der Waals energy and electrostatic energy. The van der Waals energy was calculated using the Lennard-Jones potential and the electrostatic energy was calculated using Coulomb's law. These calculations help determine the stability and adsorption of cyclodextrin on the surface of the nanostructures in the presence of different solvents.

Cell Viability assay using CCK-8

The cell viability assay was conducted using the “Cell Counting Kit 8” (CCK-8) from Sigma-Aldrich Chemie GmbH (Taufkirchen, Germany) to assess the effects of the synthesized materials on two distinct cell lines: A549 and HBE cell lines. Cells were cultured in Dulbecco’s Modified Eagle Medium (DMEM), while 16HBE14o- cells were maintained in Minimum Essential Media (MEM) supplemented with 10% fetal bovine serum, penicillin/streptomycin, and GlutaMAX or Glutamine (all obtained from Gibco BRL, Eggenstein, Germany). Cells were passaged every 3 to 4 days upon reaching 70% to 90% confluency. For the assay, cells were seeded in 96-well plates at a density of 5 × 10^5^ cells in 90 µl of cell culture medium per well, and incubated overnight at 37^°^C in a 5% CO₂ atmosphere. To account for background absorbance, 90 µl of cell culture medium without cells was added to the outer wells of the plate. The following day, serial dilutions of the compounds were prepared in Milli-Q water, and 10 µl of each concentration was added to three wells containing cells, as well as to one outer well without cells. Control groups included wells treated with 1% SDS, solvent-treated (Milli-Q water), and non-treated cells. The plates were incubated for an additional 24 hours at 37^°^C before adding 10 µl of CCK-8 solution to each well. After approximately 3 hours of incubation, absorbance was measured at 450 nm (measurement wavelength) and 650 nm (reference wavelength) using a plate reader (Infinite Pro 200, Tecan Group Ltd., Männedorf, Switzerland). Each assay was performed in triplicate.

Cell viability was determined by calculating the corrected absorbance, which is the absorbance at 450 nm minus that at 650 nm. The background absorbance from wells without cells was subtracted from the corrected absorbance of each triplicate to obtain the final values. The cell viability percentage for each treatment was expressed relative to the non-treated control, which was assigned a value of 100% viability. Results are presented in bar charts representing the mean cell viability from the three replicates, with standard deviations included for statistical relevance.

Ethical Approval/Written Consent statement


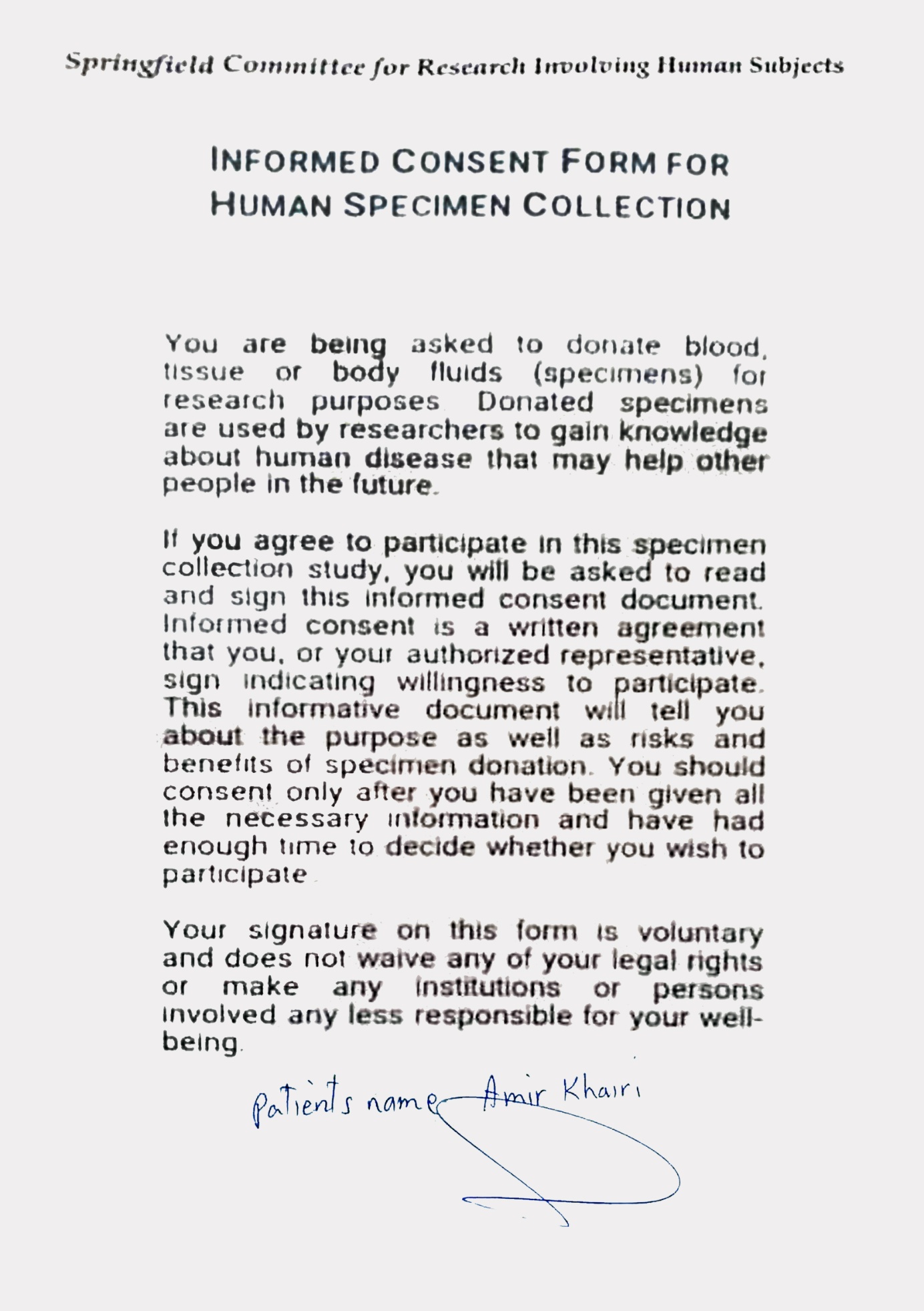


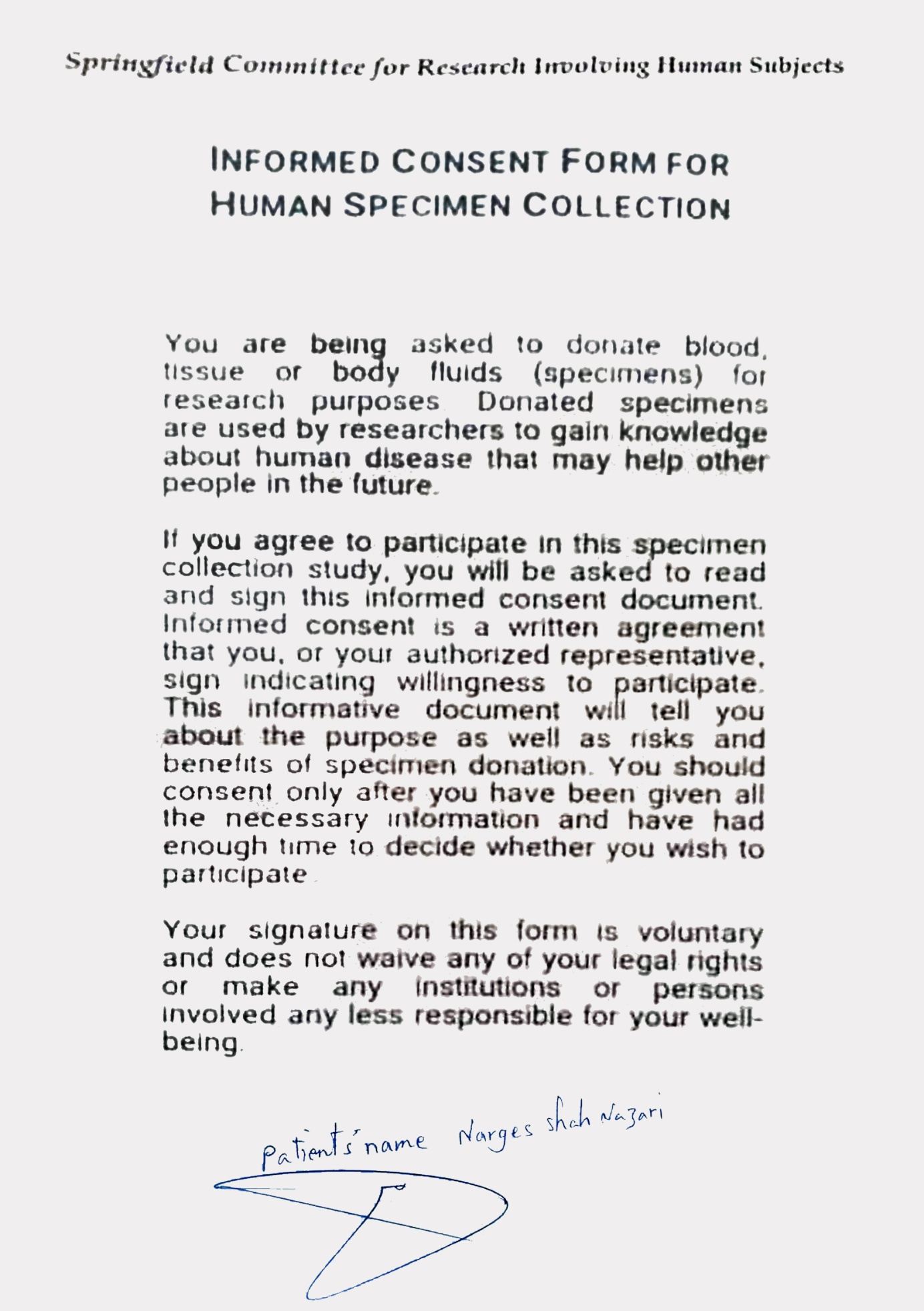


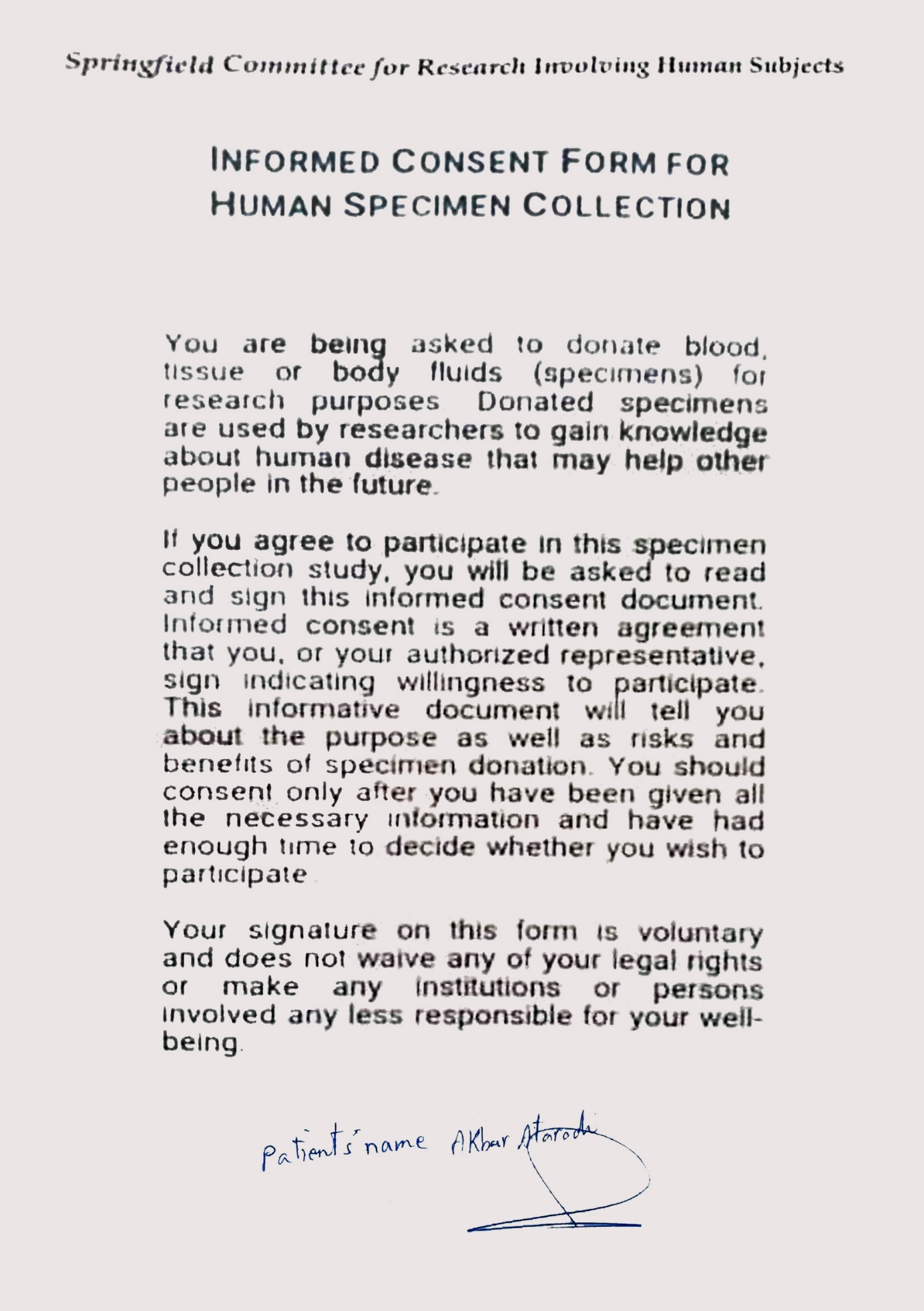


# Synthesis of heptakis (6-deoxy-6-iodo)-b-cyclodextrin (I_7_-β-CD)

I_7_-*β*-CD was synthesized according to reported procedures in literature with a slight modification ^1^. Triphenylphosphine (pph_3_) (8.0 g, 30.5 mmol) was dissolved in dry DMF (34 ml) and iodine (8.0 g_,_ 31.4 mmol) was slowly added over 30 min to the obtained solution. Then, *β*-cyclodextrin (2.0 g, 1.76 mmol) was added to the reaction mixture and it was stirred at 70 °C for 24 h under nitrogen. Afterwards, pH was adjusted at 9-10 using sodium methoxide in methanol (3 M, 75 mL). The brown precipitate was separated by centrifugation and then washed several times with methanol until no discoloration of the solvent was observed. Heptakis (6-deoxy-6-iodo)-b-cyclodextrin (1.8 g, 90%) was obtained as a white powder after drying under vacuum.

^1^H NMR (400 MHz, DMSO-d_6_, d, ppm): 6.01 (d, 7H), 5.90 (d, 7H), 4.97 (d, 7H), 3.78 (bd, 7H), 3.55–3.65 (m, 14 H), 3.33–3.45 (m, 14 H), 3.27 (t, 7H).

# Synthesis of heptakis (6-azido-6-deoxy)-*β*-cyclodextrin ((N_3_)_7_-β-CD)

(N_3_)_7_-*β*-CD was synthesized according to reported method in literature ^1^. I_7_-*β*-CD (1.8 g, 0.94 mmol) was dissolved in dry DMF (63 ml) and sodium azide (NaN_3_) (0.81 g, 12.4 mmol) was added to the obtained solution and stirred for 72 h at 80 °C under nitrogen. The reaction mixture was poured in cold distilled water and sediment was washed with water. The product was obtained as a white solid powder after drying under vacuum (1.26 g, 70%).

^1^H NMR (400 MHz, DMSO-d_6_, d, ppm): 5.86 (d, 7H), 5.73 (d, 7H), 4.89 (d, 7H), 3.69–3.77 (m, 14H), 3.55–3.60 (m, 14H), 3.29–3.36 (m, 14H).

# Synthesis of two-dimensional polycyclodextrins on colloidal templates via click chemistry (2D-CDs)

Reduced graphene oxide (rGO) and boron nitride were used as colloidal templates for the preparation of 2D-CDs.

rGO was synthesized according to reported method in literature ^2^. Briefly, graphene oxide (GO) was prepared by Hummer’s method and then reduced by heating in a suitable furnace.

To prepare Boron Nitride sheets: 2 gram of powdered boron nitride was poured into 100 ml of DMF and sonicated for 6 hours to obtain a suspension. Then it was left at room temperature for 24 h and supernatant was decanted afterwards. The stable suspension was used for the polymerization ^3^.

rGO (2.0 g) was dispersed in DMF (100 ml) and sonicated at room temperature for 1 h. Then, (N_3_)_7_-*β*-CD (1.4 g, 1.06 mmol) was slowly added to the reaction mixture and stirred at room temperature for few minutes and then tripropargylamine (0.2 ml, 1.4mmol) was added to the mixture. Afterwards, sodium ascorbate (0.64 g, 3.2 mmol) and copper sulfate (0.07 g, 0.43mmol) were dissolved in water (2 ml) and stirred at room temperature for few minutes until the color of the solution turns from dark brown to yellow and it was added to the rGO dispersion. The reaction mixture was stirred under N_2_ for 48 h at 60 °C. Cooling down the reaction mixture, the reduced graphene oxide (rGO) and boron nitride (BN) templates precipitated in the reaction medium due to the absence of functional groups on their surfaces. To remove all colloidal templates, the reaction mixture was cooled down and centrifuged at 6000 rpm several times until any sediment was observed. Due to the low dispersibility of templates, they were precipitated and product remained in supernatant. Supernatant was dialyzed against water containing EDTA (0.1g, 0.3mmol), distilled water for 48 h, DMF for 48 h, again distilled water for 48 h and methanol for 24 h. Product was obtained as a light-yellow powder (0.63g, 45%) ^4^. Polymerization of (N_3_)_7_-*β*-CD using tripropargylamine by click reaction on the BN template was performed at the same conditions.

# Synthesis of sulfated two-dimensional polycyclodextrin (2D-CDSs)

Two-dimensional polycyclodextrin (2D-CDs) (0.3 g, 0.19 mmol) was dispersed in DMF (20 ml) and sonicated for 10 minutes. Pyridine sulfur trioxide (3.0 g, 18.84 mmol) dissolved in dry DMF (20 ml) was added to the reaction mixture and stirred for 24 hours at 60 °C. Then pH was adjusted at 7 using NaOH (1 M). The product was dialyzed for 24 hours in saturated NaCl solution and then for 72 hours in distilled water by dialysis bag (MWCO 2 kDa). After lyophilization, the product was obtained as a white powder. (0.23 g, 75%) ^5^.

# Interactions between 2D-CDs and atherosclerotic plaques

Plaque samples were obtained from Tehran Heart Center Hospital approved by the hospital's ethics research committee. Written informed consent was obtained from three patients aged 50-63 years with atherosclerosis, who underwent standard endarterectomy surgery. Plaques were removed from patients carefully to keep their structural integrity and frozen in a buffer solution at -20 °C.

After splitting plaques into equal parts, they were divided into two groups containing polymer treated and untreated as the control group in PBS buffer. The plaques were transferred to a container containing 5 mg/ml of polymer in PBS and incubated for certain time frames at 37 °C. After each time frame, a piece of plaque was taken out for analysis and a new dose of polymer was added to the medium. The control group was incubated in PBS for 14 days at 37 °C and a piece of plaque was taken out and studied at different time frames.

# Investigation of the loading capacity of 2D-CDs for cholesterol and their ability to suck out this compound from plaques by HPLC

A Shimadzu HPLC device (SCL-10AVP) HPLC equipped with a C18 column (250×4.6 mm, 5 µm), UV–vis detector (SPDM10Avp), quaternary pump (LC-10ATvp), vacuum degasser and system controller (SCL10Avp), manual injector with a 10 µL sample loop was used. Shimadzu’s LabSolutions software was used for the process of chromatographic data. Identification of cholesterol was performed using mobile phase consisting of acetonitrile and isopropanol solvent, HPLC grade, with a ratio (v:v) of 30:70. The column oven, flow rate and UV detector were set at ambient temperature , 1.0 mL/min and 239 nm, respectively. all samples were repeated three times(n=3).

# Determination of loading capacity:

Dispersions of 2D-CDs with 1, 3, 5 mg/ml concentrations in PBS were prepared. Then, 5 mg/ml of cholesterol in methanol was added to these dispersions and mixture was left at 37 °C for 14 days. Mixture was filtered and the obtained solution was injected to HPLC to determine the remained unloaded cholesterol in solution. A calibration curve obtained using solutions of cholesterol (1-5 mg/l in methanol) was used for the determination of loading capacity of polymers.

# Sucking out cholesterol from plaques

Plaques were collected from three different patients with atherosclerosis and incubated separately with 5 mg/ml of 2D-CDs in PBS for 14 days at 37 °C. Afterwards, the supernatant was filtered and used for the injection into the HPLC and determination of the amount of cholesterol.

# Characterizations


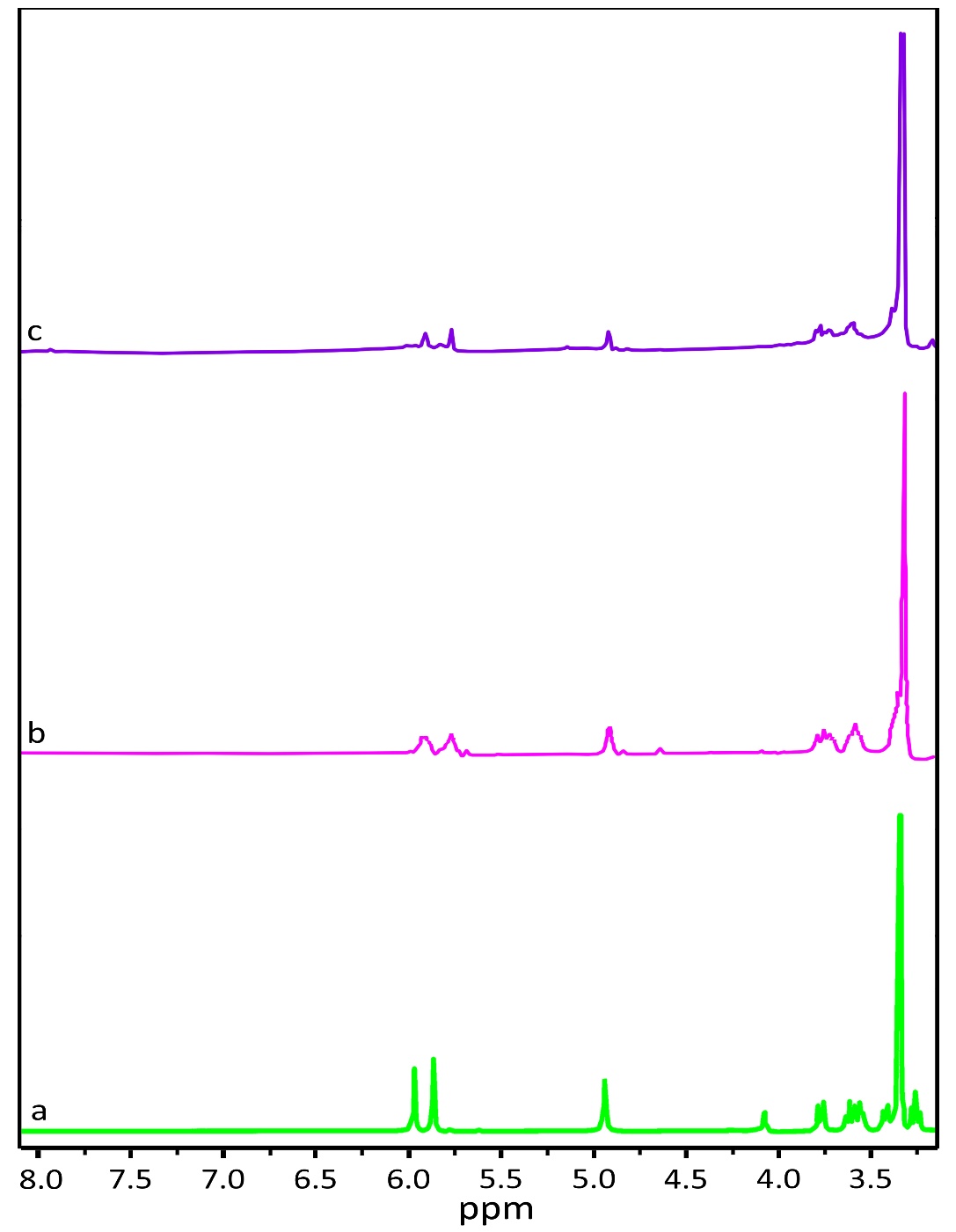


## **Figure S1.** ^1^H NMR spectra of (a) *β*-cyclodextrin, (b) heptakis-(6-deoxy-6-iodo-)-*β*-cyclodextrin, (c) heptakis-(6-azido-6-deoxy)-*β*-cyclodextrin.

*Size, morphology and composition of templates,* 2D-CD*s and samples*

**
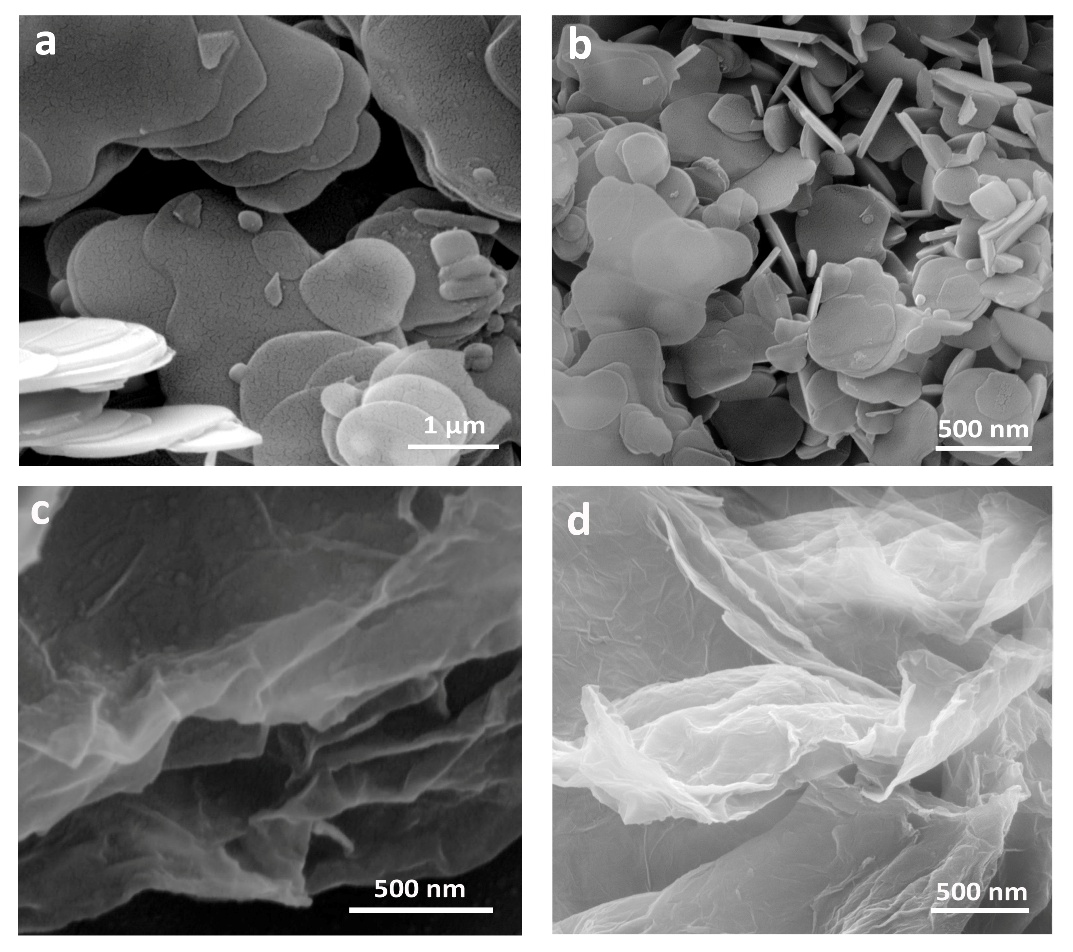
**

## **Figure S2.** SEM images of (a, b) BN and (c, d) rGO that were used as templates for the synthesis of 2D-CDs.

# Self-assembly of heptakis (6-azido-6-deoxy)-*β*-cyclodextrin ((N_3_)_7_-β-CD) on highly oriented pyrolytic graphite

In order to prove the ability of rGO template for the confining (N_3_)_7_-*β*-CD monomers in a monolayer assembly, their interactions with the freshly cleaved highly oriented pyrolytic graphite (HOPG) were investigated. This gives us more information regarding the mechanism of the synthesis of 2D-CDs on rGO template.

It has been previously reported that HOPG, due to its low surface energy, allows self-arrangement and assembly of the small molecules on its surface in a solid– liquid interface. Self-assembled monolayers or sub-monolayers are detectable by the Scanning force microscopy in Tapping (SFM-TM) or Quantitative imaging modes (SFM-QI). Additionally, scanning tunneling microscopy (STM) allows higher laterally resolved investigation of the self-assembled monolayers in solid-liquid interfaces. Here, we demonstrated that (N_3_)_7_-*β*-CD dispersed in N, N-Dimethylformamide (DMF) solvent at very low concentrations 0.1 mg/ml is able to form self-assembled sheet-like structures at solid-liquid interface of freshly cleaved HOPG substrate.

Scanning force microscopy in tapping and quantitative imaging modes were used to investigate the formation of 2D self-assembled (N_3_)_7_-*β*-CD in N, N-Dimethylformamide (DMF) solvent over the freshly cleaved HOPG. A solution of the (N_3_)_7_-*β*-CD (0.1mg/ml) in DMF was prepared and dropped (50 µl) onto a freshly and mechanically cleaved HOPG (Momentive Performance, ZYA quality). For SFM-QI and SFM-AC (Tapping mode) measurements a JPK Nanowizard 3 (JPK, GmbH) was used. All measurements were carried out using either a 1.7 N/m or a 0.35 N/m cantilevers made of silicon with silicon nitride coatings from Olympus and Bruker, respectively. Cantilever sensitivities were calibrated using the thermal tuning method generally used for soft cantilevers. SFM-QI measurements were carried out at a scanning rate of 5-8 minutes per image with 600 to 1000 sampling rates. SFM measurements were carried out for freshly and mechanically cleaved HOPG substrate in ambient conditions as a reference and after placement of the (N_3_)_7_-*β*-CD dispersed in DMF as a droplet. A density of 944.0 kg/m³ and viscosity of 889.0 µPa·s was considered for the in liquid DMF measurements for calculation of the cantilever thermal noise.

Figure S3 demonstrates the typical overview of a freshly cleaved HOPG surface at ambient conditions as measured by SFM-QI and after addition (N_3_)_7_-*β*-CD in DMF droplet. A thin layer of (N_3_)_7_-*β*-CD is created over the HOPG surface which does not follow a certain shape or size. The height of the islands formed on the flat terraces of HOPG is 0.8 nm, which is close to the height of *β*-CD (0.78 nm) in literature. We attribute the small difference between the theoretical height (0.78 nm) and the measured value in this study (0.8 nm) to the azide covalently attached to the *β*-CD.

Furthermore, it was observed that the process of self-assembly was not instantaneous and formation of the (N_3_)_7_-*β*-CD islands over the HOPG required 5-20 minutes, regardless of scanning by SFM. We attribute this to the fact that the fluid dynamic in homogeneity in the DMF solvent during the approach and retract processes can cause distortion and disruption of the self-assembled mono and sub monolayers of the materials in solid-liquid interfaces.

Inspired by the ability of HOPG to confine and organize (N_3_)_7_-*β*-CD monomers in DMF interface, we decided to use rGO to support the lateral crosslinking of these monomers by click reaction. It is worth noting that the HOPG used in this work is high quality compound with minimal structural defects or oxygen containing functional groups.


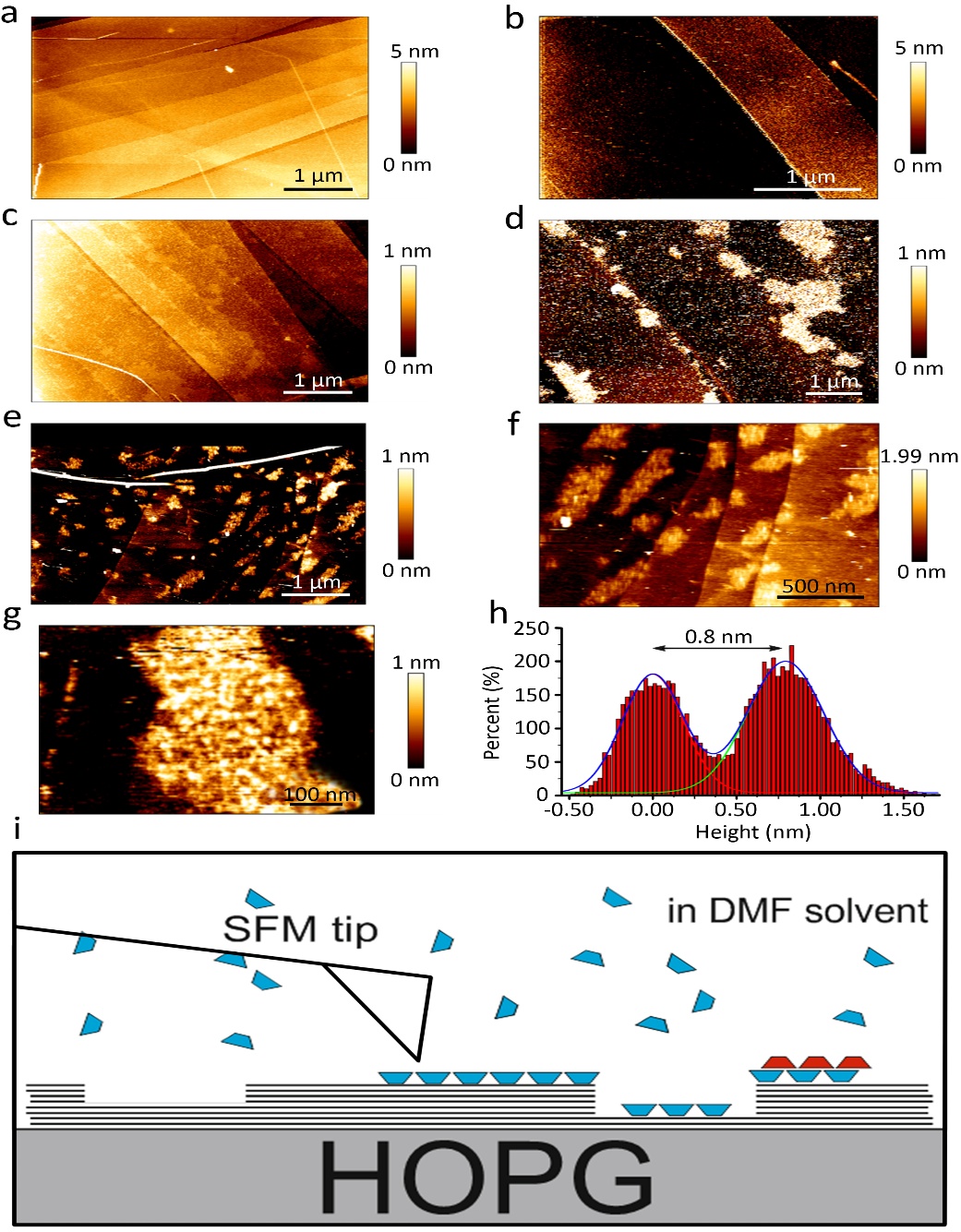


## **Figure S3**. (a and b) SFM-QI height image of the freshly cleaved HOPG surface acquired at ambient conditions (23 ^o^C and 32% relative humidity). (c) SFM-QI height image of (N_3_)_7_-*β*-CD) molecules at DMF-HOPG interface. (d) Close up view of the center of figure (c). (e) SFM-AC height image of the ((N_3_)_7_-*β*-CD) molecules at DMF-HOPG interface. (f) Close up of the self-assembled layers over the HOPG surface. (g) Demonstration of the self-assembled layer of ((N_3_)_7_-*β*-CD) over HOPG surface in DMF solvent and (h) the height distribution of the HOPG and (N_3_)_7_-*β*-CD) layer. (i) Schematic view of in solvent (liquid) SFM measurement of the self-assembly of (N_3_)_7_-β-CD over HOPG layered crystal.


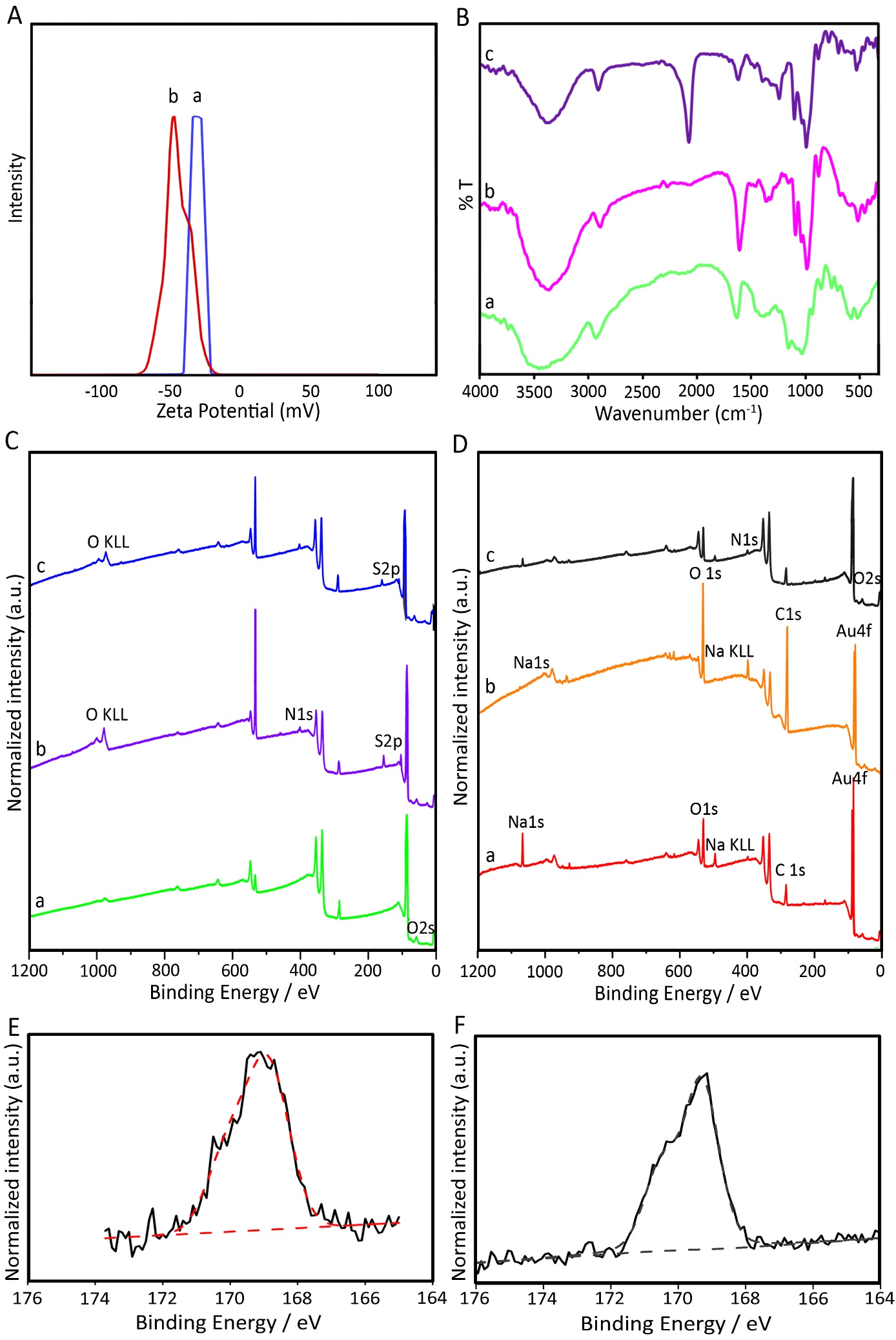


## **Figure S4.** (A) Zeta potential diagram of (a) 2D-CDs and (b) 2D-CDSs. The surface charge of 2D-CDSs was slightly negative, due to the presence of high amount of nitrogen. After sulfation the negative surface charge increased to -44 mV. (B) IR spectra of precursors of 2D-CDs including (a) *β*-cyclodextrin, (b) heptakis (6-deoxy-6-iodo)-*β*-cyclodextrin (I_7_-*β*-CD) and (c) heptakis (6-azido-6-deoxy)-*β*-cyclodextrin (N_3_)_7_-*β*-CD. Based on XPS spectra, 2D-CDs polymers were mainly composed of carbon, oxygen and nitrogen, as it was expected. Survey XPS spectra of (C) (a) *β*-CD, (b) heptakis (6-azido-6-deoxy)-*β*-cyclodextrin (N_3_)_7_-*β*-CD, (c) 2D-CDs synthesized on BN templates. (D) (a)2D-CDSs synthesized on BN templates, (b) 2D-CDs and (c) 2D-CDSs synthesized on rGO templates respectively. Highly resolved S2p XPS spectra of 2D-CDSs synthesized on: (E) BN and (F) rGO templates. Highly resolved S2p XPS spectrum of 2D-CDSs synthesized on rGO template showed a doublet peak at 169.3 eV, confirming successful sulfation of 2D-CDs. Appearance of S2p peak at 168.9 eV after sulfation of 2D-CDs synthesized on BN template confirms successful production of 2D-CDSs. However, for both non-sulfated samples no sulfur is detected.

##
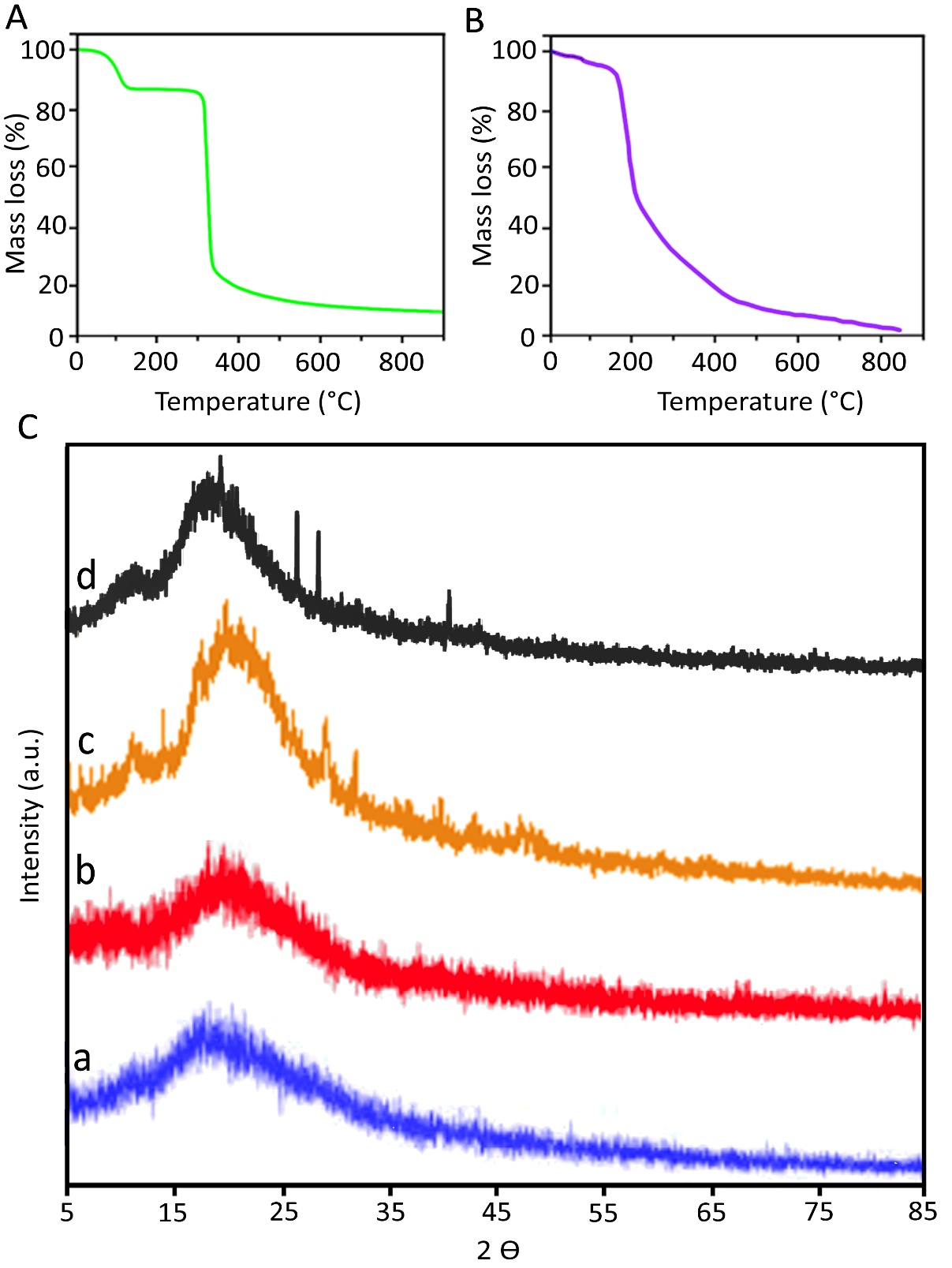


## **Figure S5.** TGA thermogram of (A) *β*-cyclodextrin, demonstrating a weight loss (14%) at 120 °C, due to the evaporation of water and a main weight loss (77%) at 350 °C, assigned to destruction of it backbone. **(**B) heptakis (6-azido-6-deoxy)-*β*-cyclodextrin ((N_3_)_7_-*β*-CD, demonstrating a main weight loss (51%) at 220 °C. Decreasing the thermal stability of cyclodextrin after azidation can be assigned to the decomposition of azide functional groups and releasing nitrogen gas. (C) Powder XRD diffractograms of (a) 2D-CDs and (b) 2D-CDSs synthesized on BN template respectively, (c) 2D-CDs and (d) 2D-CDSs synthesized on rGO template respectively.

# Toxicity of the synthesized materials

**
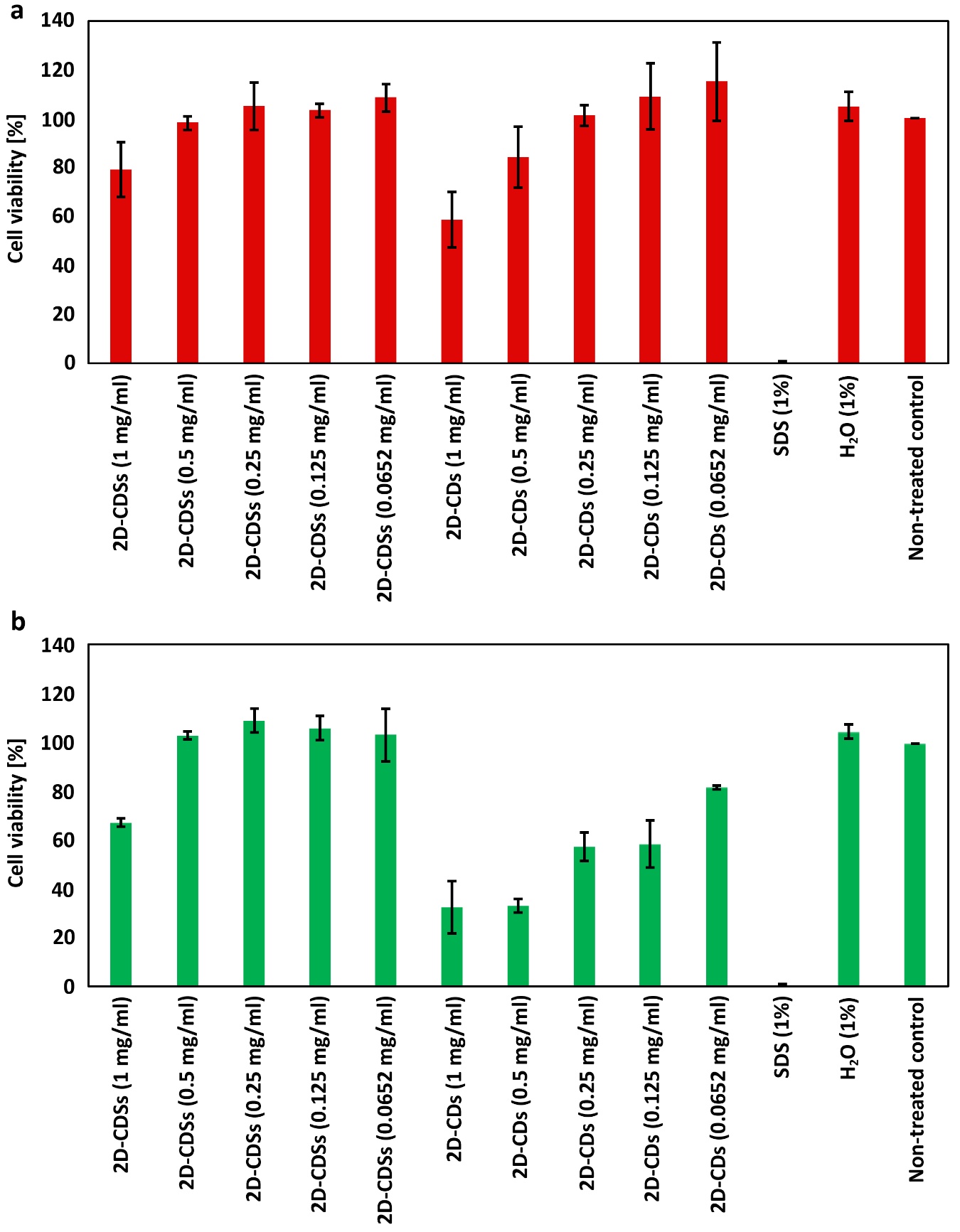
**

## **Figure S6**. The cytotoxicity of 2D-CDs and 2D-CDSs, synthesized on rGO template, against A549 (a) and HBE (b) cell lines using CCK8 assay. Toxicity of 2D-CDs was higher than that for 2D-CDSs and varied by changing the cell line. The higher toxicity of 2D-CDs can be assigned to its lower solubility and big agglomerations. 2D-CDSs showed a high biocompatibility against both cell lines and didn’t show significant toxicity up to 0.5 mg/ml.

# Loading and sucking out cholesterol from plaques by 2D-CDSs

To calculate loading capacity of polycyclodextrin for cholesterol, the freeze-dried 2D-CDSs (1 mg, at -50 °C for 24h) was added to a falcon tube containing different concentration of cholesterol in methanol (2 ml). The sample was sonicated at 37 °C for 24 h. Then, it was left at room temperature for one hour. Then, mixture was centrifuged at 10000 rpm and supernatant was filtered using 45 μm PDVF filter. For the determination of the loaded cholesterol, the supernatant was monitored by HPLC and the absorbance of the samples was recorded at 205 nm.

For the calibration curve, a stock standard solution of cholesterol (5 μg/ml) was prepared by dissolving cholesterol in methanol. Different concentrations of cholesterol were prepared by diluting the stock solution with methanol. The calibration curve was obtained using different concentrations of cholesterol in the range of 1-5 μg/ml with the equation y=10656x+45632 and R^2^=0.995. Figure S7a shows the calibration curve in the investigated linear region.

The loading capacity of 2D-CDSs was calculated using the following equation:

$$Loading capacity \%=\frac{loaded cholestrol weight}{2DCDSs polymer weight}\times100$$

# Loading and sucking out cholesterol from plaques by 2D-CDSs

A stock standard solution (5 μg/ml) was prepared from cholesterol solution in methanol. Different concentrations of cholesterol were prepared by diluting the stock solution with methanol.

The calibration curve was obtained using different concentrations of cholesterol in the range of 1-5 μg/ml with the equation y=10656x+45632 and R^2^=0.995. Figure S6a shows the calibration curve in the investigated linear region.

*Investigation of the ability of 2D-CDSs for multivalent interactions with plagues and sucking cholesterol from their structures out*


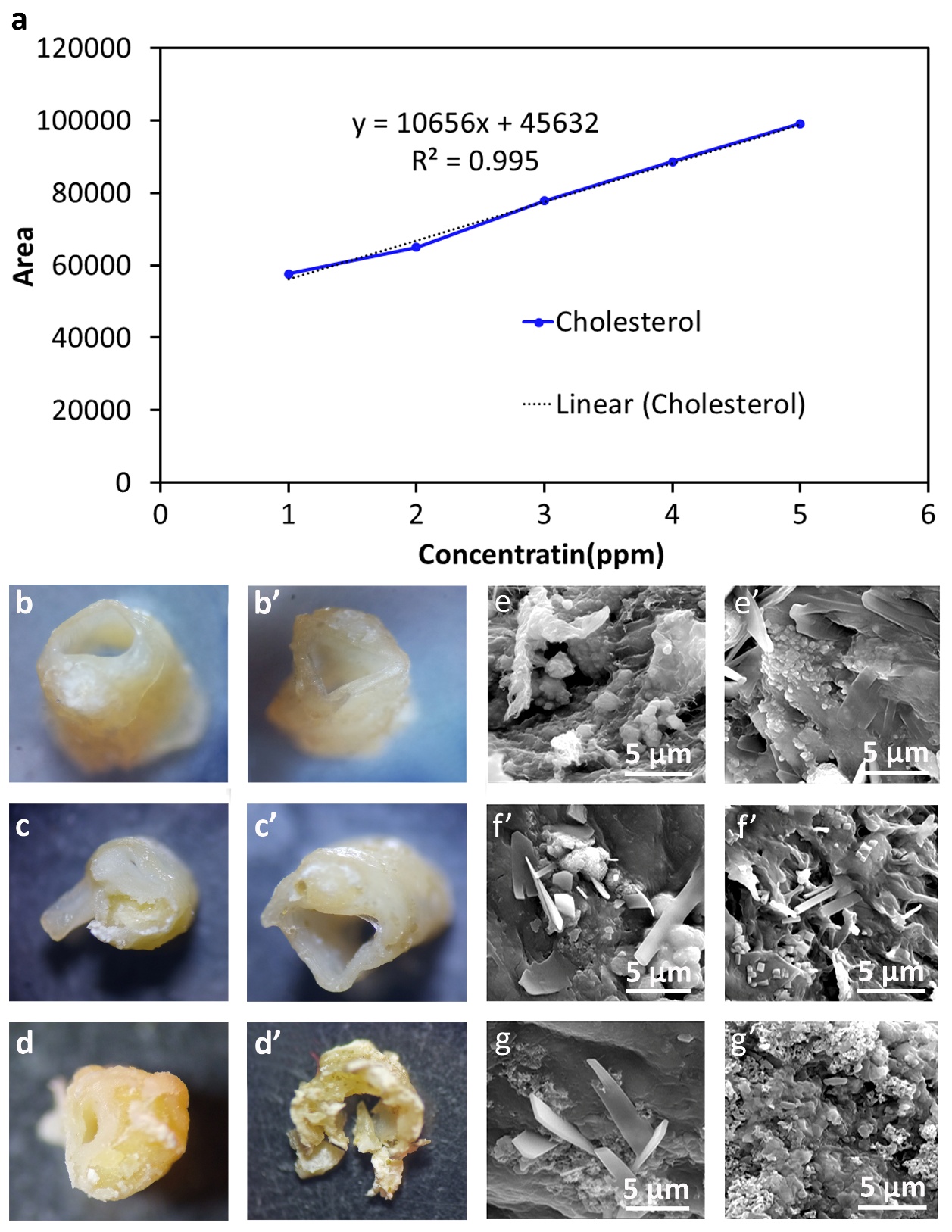


## **Figure S7**. (a) Calibration curve of obtained using different concentrations of methanol solution of cholesterol in the range of 1-5 μg/ml. Evaluating of the (b) optical microscopy and (c) SEM images of pieces of plaques in the absence (i-iii) and presence (i’-iii’) of 2D-CDSs after 1, 3 and 14 days, clearly showed the ability of both materials to extract cholesterol from plaques. Plaques were broken down after two weeks interactions with 2D-CDSs.


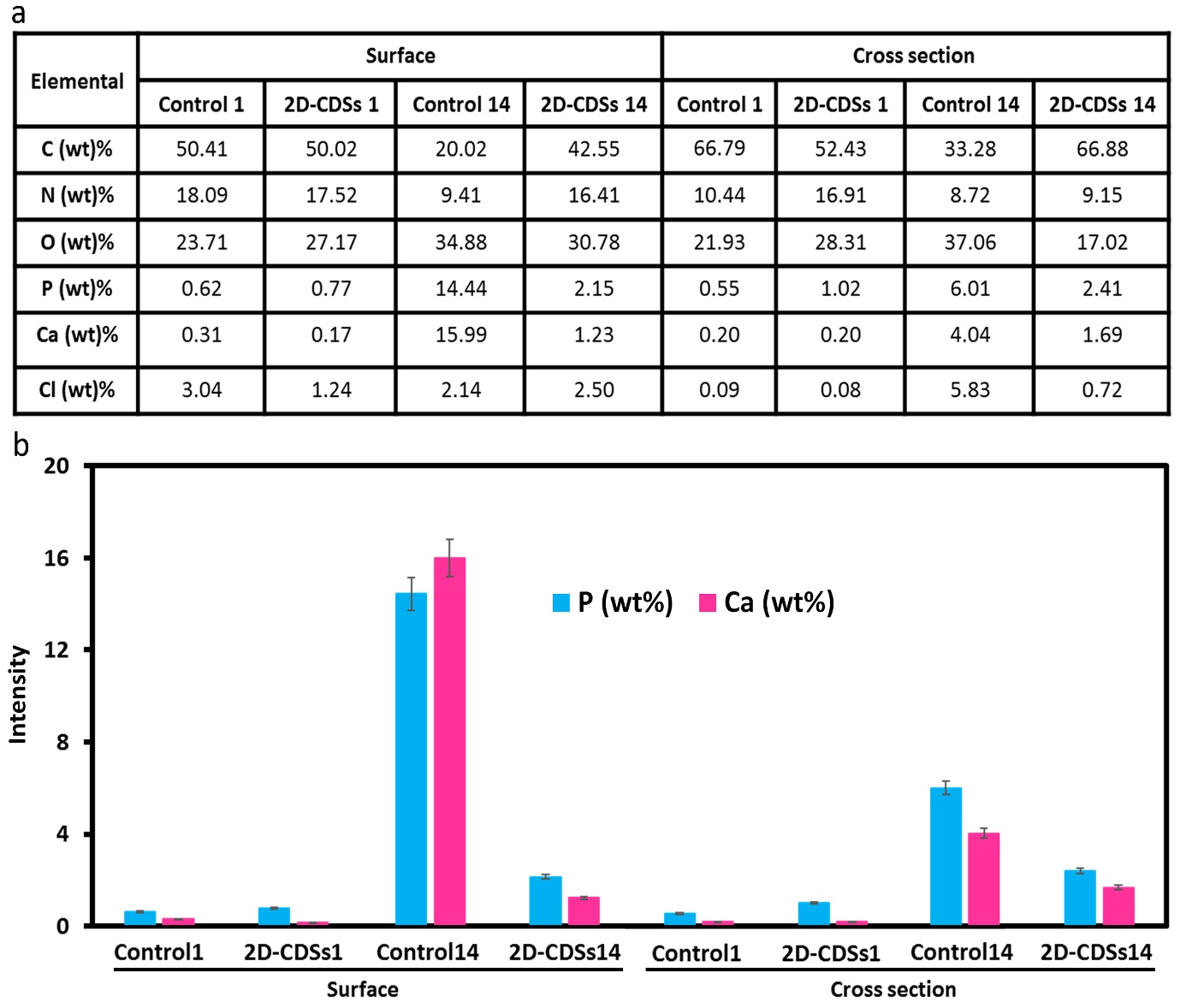


## **Figure S8.** (a) Evaluation of the composition of plaques in the absence (control) and presence of 2D-CDSs after and one and 14^th^ days of incubation from the surface and cross section of plaques by EDX. (b) Composition of plaques in terms of calcium and phosphorus measured in the absence (control) and presence of 2D-CDSs after and one and 14^th^ days of incubation from the surface and cross section of plaques by EDX.

After preparing the samples, they were injected into the HPLC device to measure the amount of adsorbed cholesterol.

Retention time (RT) for cholesterol according to the spiked sample was 2.9/min (Figure S8).

Based on the chromatograms of standard samples (cholesterol), peaks of cholesterol/2D-CDSs and plaque/2D-CDSs indicated the amount of cholesterol loaded by the 2D-CDSs. The capacity of different concentrations of 2D-CDSs (1, 3, 5 mg) to load primary cholesterol (5 mg/ml) was obtained as 0.66, 1.07, and 1.62 mg, respectively (Figure 5a). Due to the increase in polymer concentration, the amount of cholesterol absorption also increases. The amount of cholesterol separated from 1 mg of plaques (A, B and C) treated with 1 mg of 2D-CDSs showed 9.04, 9.05, and 9.07×10^-2^ mg of cholesterol (Figure 5b).

# The loading capacity was calculated by HPLC using this equation:

$$Loading capacity \%=\frac{loaded cholestrol weight}{2DCDSs polymer weight}\times100$$

$$Loading capacity \%=\frac{0.665 \mathrm{mg}}{1 \mathrm{mg}}\times100=66.5\%$$

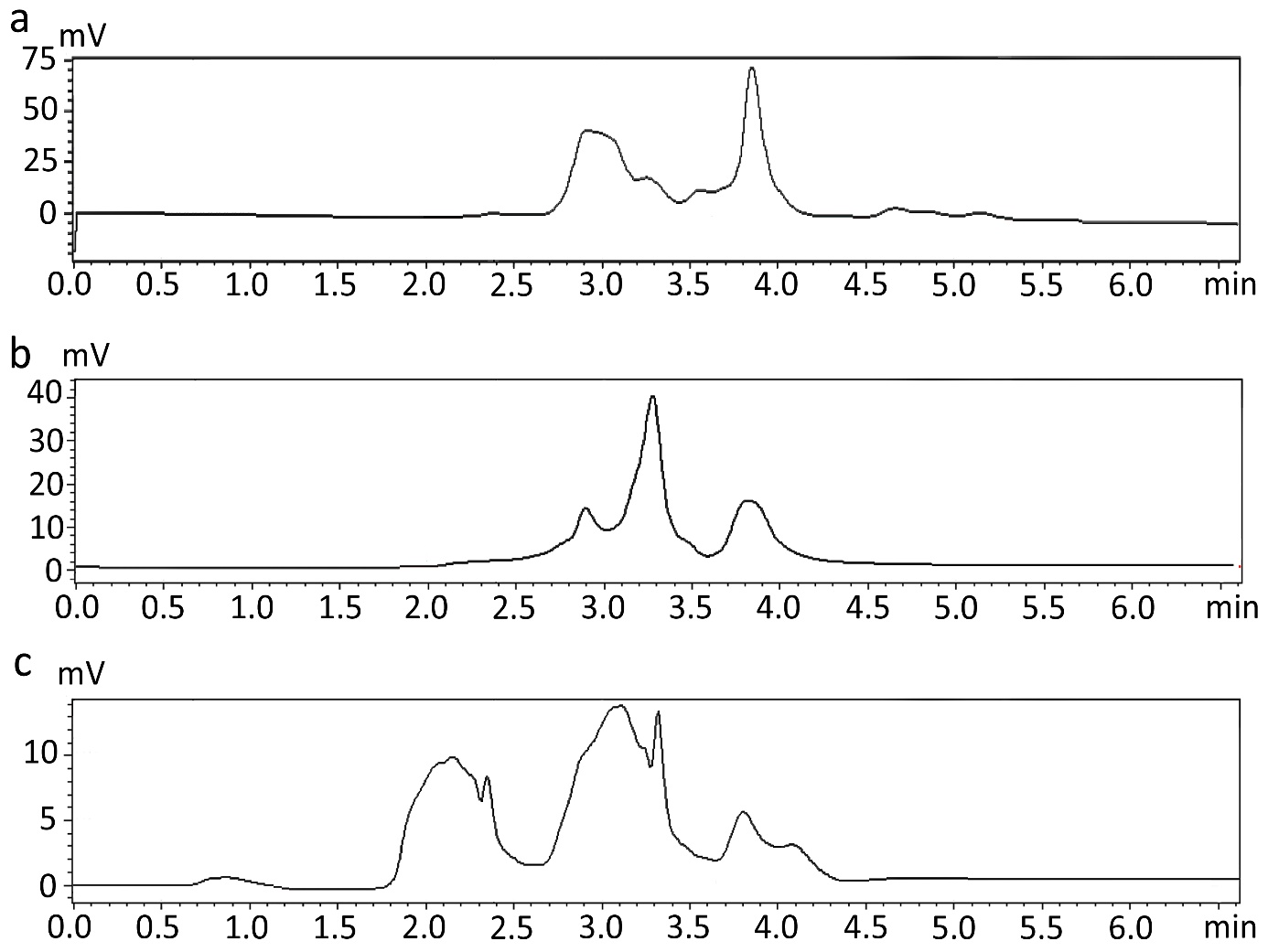


## **Figure S9.** (a) Evaluation of the area under the curve of stock concentration of cholesterol (1-5 µg/ml). Investigation of the host-guest interaction between (b) cholesterol (5 mg/ml) and (c) plaque (1 mg) different concentration of 2D-CDSs (1, 3 and 5 mg/ml) using HPLC.

# Estimation of the molecular weight of 2D-CDSs synthesized using graphene template

Outer diameter of β-cyclodextrin is 1.54 nm ^6^. Considering azide segments and also tripropargylamine crosslinker, the size of each unit of 2D-CDSs will be around 2 nm. Also, molecular weight of each unit, considering seven azide groups and three propargylamine crosslinkers, is 1815. 2D-CDSs sheets with an average dimension around, 4 × 10^4^ nm^2^ are consisting 2 × 10^4^ unite and based on that the molecular weight of each sheet is around 5.78 × 10^6^. Using this molecular weight,10 μg/ml solution of 2D-CDSs is almost 1.14 nM.

**Movie S1:**

Movie S1 shows the tomogram of a 2D-CD sheet synthesized on rGO template. Interactions between monomers and rGO in DMF are strong enough to inhibit their mobility for a long-range polymerization. Thus, their lateral size of sheets was limited to few hundreds of nanometers.

# References

[1] S. Srinivasachari, K. M. Fichter, T. M. Reineke, *J. Am. Chem. Soc.* **2008**, 130, 4618.

[2] S. Setiadji, B. W. Nuryadin, H. Ramadhan, C. D. D. Sundari, T. Sudiarti, A. Supriadin, A. L. Ivansyah, Preparation of Reduced Graphene Oxide (rGO) Assisted by Microwave Irradiation and Hydrothermal for Reduction Methods. 2018, IOP Conf. Ser.: *Mater. Sci. Eng.* 434 012079.

[3] J. Hassan, M. Ikram, A. Ul-Hamid, M. Imran, M. Aqeel, S. Ali, *Nanoscale Res. Lett.* **2020**, 15, 1.

[4] R. Kabiri, H. Namazi, *J. Nanoparticle Res.* **2014**, 16, 1.

[5] F. Reisbeck, A. Ozimkovski, M. Cherri, M. Dimde, E. Quaas, E. Mohammadifar, K. Achazi, R. Haag, *Polymers* **2021**, 13, 982.

[6] D. Noreña-Caro, M. Alvarez-Láinez, *IJIDeM* **2016**, 10, 153.
